# Musculoskeletal Health and Work: Development and Internal–External Cross-Validation of a Model to Predict Risk of Work Absence and Presenteeism in People Seeking Primary Healthcare

**DOI:** 10.1007/s10926-024-10223-w

**Published:** 2024-07-04

**Authors:** Lucinda Archer, George Peat, Kym I. E. Snell, Jonathan C. Hill, Kate M. Dunn, Nadine E. Foster, Annette Bishop, Danielle van der Windt, Gwenllian Wynne-Jones

**Affiliations:** 1 School of Medicine, Keele University, Staffordshire, ST5 5BG UK; 2 Institute of Applied Health Research, University of Birmingham, Edgbaston, Birmingham, B15 2TT UK; 3 National Institute for Health and Care Research (NIHR) Birmingham Biomedical Research Centre, Birmingham, UK; 4 Centre for Applied Health and Social Care Research (CARe), Sheffield Hallam University, Sheffield, S10 2BP UK; 5 Surgical Treatment and Rehabilitation Service (STARS) Education and Research Alliance, The University of Queensland and Metro North Hospital and Health Service, St Lucia, QLD Australia

**Keywords:** Work absence, Prognosis, Prognostic model, Musculoskeletal pain, Primary care

## Abstract

**Purpose:**

To develop and validate prediction models for the risk of future work absence and level of presenteeism, in adults seeking primary healthcare with musculoskeletal disorders (MSD).

**Methods:**

Six studies from the West-Midlands/Northwest regions of England, recruiting adults consulting primary care with MSD were included for model development and internal–external cross-validation (IECV). The primary outcome was any work absence within 6 months of their consultation. Secondary outcomes included 6-month presenteeism and 12-month work absence. Ten candidate predictors were included: age; sex; multisite pain; baseline pain score; pain duration; job type; anxiety/depression; comorbidities; absence in the previous 6 months; and baseline presenteeism.

**Results:**

For the 6-month absence model, 2179 participants (215 absences) were available across five studies. Calibration was promising, although varied across studies, with a pooled calibration slope of 0.93 (95% CI: 0.41–1.46) on IECV. On average, the model discriminated well between those with work absence within 6 months, and those without (IECV-pooled C-statistic 0.76, 95% CI: 0.66–0.86). The 6-month presenteeism model, while well calibrated on average, showed some individual-level variation in predictive accuracy, and the 12-month absence model was poorly calibrated due to the small available size for model development.

**Conclusions:**

The developed models predict 6-month work absence and presenteeism with reasonable accuracy, on average, in adults consulting with MSD. The model to predict 12-month absence was poorly calibrated and is not yet ready for use in practice. This information may support shared decision-making and targeting occupational health interventions at those with a higher risk of absence or presenteeism in the 6 months following consultation. Further external validation is needed before the models’ use can be recommended or their impact on patients can be fully assessed.

**Supplementary Information:**

The online version contains supplementary material available at 10.1007/s10926-024-10223-w.

## Introduction

Musculoskeletal disorders (MSDs) are a common cause of disability with the Global Burden of Disease projects estimating that they account for 21% of all years lived with disability [1]. It is reported that globally 1.7 billion people experiencing MSDs would benefit from rehabilitation, with low back pain the most prevalent condition in the majority of countries [2]. In the UK it is estimated that 20.3 million people (almost one third of the population) have an MSD [3] and in any one year 20% of the adult population will consult their general practitioner with an MSD [4]. Musculoskeletal Disorders are among the most costly conditions, with estimates from Australia suggesting they cost more than both cardiovascular disease and cancer [5] and from the UK suggesting they account for the third largest area of NHS healthcare spending [3]. The overall economic burden is even higher when indirect costs such as sick leave, loss of productivity, and early retirement are considered [3]. Musculoskeletal Disorders can also lead to significant impacts on individuals, such as significant pain, reduced mobility, poor quality of life, and reduced abilities for employment [6, 7].

Roughly 60% of workers report one or more MSDs in the past 12 months (data from 2015), an estimate that varies by country, sector, occupation, and individual sociodemographic characteristics [8]. Musculoskeletal Disorders have a significant cost in terms of employment with these costs extending to individuals, employers, and society. Not only are people with MSDs less likely to be in work but they are also more likely to retire early when compared to people without a long-term health condition [3]. The impact of MSDs on work can be defined in terms of presenteeism (or performance in the workplace) and absence from the workplace. Musculoskeletal Disorders are one of the leading causes of reduced performance and sickness absence and it is estimated that the 100 million people in Europe affected by MSDs account for half of all absences from work and 60% of permanent work incapacities [9]. In the UK alone, an estimated 28.4 million working days per year are lost as a result of MSDs [10]. Furthermore, costs to productivity associated with sick leave caused by MSDs in Europe are estimated at €5798 per person with health care costs accounting for 9.3% of these and rehabilitation accounting for 3.7% [11]. When costs of illness are considered in more detail, it is apparent that indirect costs account for the largest proportion of these, with work absence in particular contributing to this [12, 13].

Across all working ages, it is the small proportion of people going on to long-term absence who make up the majority of costs associated with absence from the workplace [13]. However, there is currently no reliable way of predicting which employees will require additional vocational advice and support and which employees will return to work quickly without this support [14]. Being able to predict which individuals are at greatest risk of work absence when they consult with their primary care clinician could inform more targeted interventions to minimise the impact of MSDs on the workforce. Evidence suggests that vocational interventions can cost-effectively support people with MSDs to work [15], however workforce constraints mean that a model of targeted provision may be the most realistic method of implementing a vocational advice service.

Previous work exploring the prediction of sickness absence has focussed on specific predictors and conditions [16–20]. The most widely used model to predict work absence is the Orebro Musculoskeletal Pain Questionnaire, and its short version [21, 22], both of which were developed in populations with back pain only. At 6-month follow-up, the longer version (including 25 items with possible scores up to 210) achieved a sensitivity of 89% with a cut-off at a score of 90, for predicting long-term absence (defined as > 30 days over 6 months). This sensitivity reduced with increasing cut-offs for total score. When used to predict short-term absences (defined as 1–30 days over 6 months), the long version of the Orebro demonstrated a sensitivity of 67% with a cut-off at a score of 90, again with sensitivity reducing for increasing cut-off scores. However, these questionnaires are required to be completed in full for each patient, which can be difficult in first contact settings, for example primary care, where time is limited. Additionally, Orebro was developed in populations with back pain only, although it is currently used in populations with other regional pain symptoms (specifically neck and shoulder). Its suitability for use in a population with more general MSDs and those with multisite pain is not clear. Furthermore, Orebro does not predict presenteeism, which may have a bigger impact on work environments than absenteeism [23].

This study aimed to establish whether prognostic models for work absence and presenteeism, developed from existing primary care cohorts and including data that can be easily accessed from electronic medical records, could accurately predict these work-related outcomes in individuals with MSDs. We examined whether work absence could accurately be predicted using a combination of a patient’s demographic details, health-related factors and work-related factors. We report on the development and internal–external cross-validation (IECV) of prediction models to estimate the probability of an individual taking any work absence within 6 or 12 months following their consultation for MSD in primary care, and presenteeism in the 6 months after their MSD consultation.

## Methods

### Data Sources and Study Population

Data from six studies (1 prospective cohort study, and 5 randomised trials, all conducted before the COVID-19 pandemic) were combined for model development, allowing variables that were common between studies to be considered as candidate predictors [15, 24–28]. All studies were based in the West-Midlands and Northwest regions of England, recruited adult patients consulting in primary care with MSD via questionnaire after consultation, and included data on work outcomes measured during the 12 months following their consultation. These studies were selected as they provided a population of working age adults reporting MSDs.

Participants of each study were eligible if they were employed at the baseline time-point and were reporting MSDs (at any location). Studies didn’t aim to identify those with inflammatory arthritic conditions but patients with inflammatory arthritis may be included if they sought healthcare for their pain. Participants could be at work or absent from work at baseline, providing they had data on both work status (employed versus other) and other work outcomes (absenteeism, presenteeism, no reported absence) at either 6 or 12 months. For prediction of 12-month outcomes, eligible participants were further required to still be in employment at 12 months following their consultation, at this point all but one study [14] reported only whether participants were in employment or not, if they were not in employment the reason for this was not ascertained. Further details on study populations are provided in Table 1.

**Table 1:** Details of study populations in the individual studies making up the individual participant data for this research, including study setting, design, and selection criteria

Study	Dates of study	Setting	Age group	Follow-up period	Study design*	Intervention description	Additional information
Benefits of Effective Exercise for knee Pain (BEEP) trial [24]	2010–2012	General practice and physiotherapy services	≥ 45	36 months	Three parallel arm RCT	Individually tailored exercise Targeted exercise adherence Control group: usual physiotherapy	Clinical diagnosis of OA Knee pain
Keele Aches and Pains Study (KAPS) [25]	2014–2016	General practice	≥ 18	6 months	Prospective cohort	Not applicable	Self-reported pain in back, neck, shoulder, knee or multisite
Stepping up the evidence for musculoskeletal Services (STEMS) [26]	2013–2013	Physiotherapy services (referred from general practice)	≥ 18	12 months	Two parallel arm pilot cRCT	Direct access to physiotherapy service Control group: usual GP-led care and referral	All musculoskeletal pain
Study of Work and Pain (SWAP) [15]	2012–2014	General practice	18–70	12 months	Two arm cRCT	Vocational advice delivered by trained staff to support return to/stay at work Control: usual GP-led care	All musculoskeletal pain
The STarT MSK pilot trial (STarT MSK-pilot) [27]	2016–2017	General practice	≥ 18	6 months	Two parallel arm pilot cRCT	Stratified care intervention for management of MSDs Control: usual GP-led care	Self-reported pain in back, neck, shoulder, knee or multisite
STarT MSK main trial (STarT MSK-MT) [28]	2018–2019	General practice	≥ 18	6 months	Two parallel arm cRCT	Stratified care intervention for management of MSDs Control: usual GP-led care	Self-reported pain in back, neck, shoulder, knee or multisite

^*^RCT randomised controlled trial, *cRCT* cluster randomised controlled trial

### Outcome Definitions

Work absence – work absence was recorded at various time-points across the six studies, using self-report questionnaires. This was phrased as “Have you taken time off work in the last XX months because of your pain problem?”, with a follow-on question asking participants to confirm the numbers of days, weeks, and months that they were absent from work. An absence event was defined as at least one day of absence.

Presenteeism – presenteeism at six months was defined using a single, self-report item in three of the six studies [26–28]. This was defined on a 0–10 scale, in response to the question “On average to what extent has your pain or related problem affected your performance at work over the past 6 months? (Please tick one box only)”, where zero indicated “Not at all” and 10 indicated “So bad I am unable to do my job”. This question was consistently phrased across all three studies, and was collected through postal questionnaire at six months follow-up.

Further details on the phrasing of self-report outcome measures are given for each study included in each of the analyses, in Appendix I – extended methods.

### Model Predictors

Clinically relevant predictors of work absence were identified from the literature (PROSPERO CRD42020219452) and through expert clinical opinion. For model development, these were compared with baseline information collected in each study, and predictors included for analysis were limited to those that were recorded in at least half of the studies recording a given outcome. Thus, 10 predictor variables were included for predictions of 6-month absence and 6-month presenteeism, including patient demographics (age, sex, job-type [(i) managerial/administrative/professional; (ii) intermediate; (iii) routine and manual]), pain features (multisite or single site pain, pain intensity at baseline, pain duration), comorbidities (anxiety/depression, any other comorbidity), work absence in previous six months and current performance at work. For prediction of 12-month absence, predictors regarding previous absence and comorbidities were not consistently recorded across studies, leaving just eight available predictors.

All predictor information was self-reported, collected through baseline questionnaires sent after consultation. Job-title was recorded as free-text and allocated to the National Statistics Socio-economic Classification operational categories (three class) by the study authors [29].

### Sample Size

The sample size for model development was fixed for each analysis due to the size of the available studies. Riley et al. 2019 criteria for the development of prediction models with binary [30] and continuous [31] outcomes were used to identify the number of predictor parameters we could include in the models. For the primary outcome of any work absence over 6 months, a total of 2179 participants (215 events) allowed consideration of up to 16 predictor parameters, while for our model to predict 6-month presenteeism, a total of 1218 participants allowed consideration of up to 24 predictor parameters. Thus, we had sufficient data to meet minimum sample size requirements to develop both models.

The sample size available to predict 12-month absence was fixed at only 408 participants (132 events). This number was insufficient to meet the requirements for the intended 11 predictor parameters, thus results should be interpreted with caution.

More detailed information on the assessment of sample size is given in extended methods in Appendix I.

### Statistical Analysis

#### Missing Data

Multiple imputation of the multilevel data was implemented using joint modelling, to impute values for missing data across studies, accounting for the clustering within the included studies while allowing for between study heterogeneity on key parameters [32, 33]. This further allowed for the imputation of two predictor variables (absence and comorbidities) in the 12-month model that were systematically missing by borrowing information across all studies (a summary of missing data is given in supplementary table S1). This approach was implemented using the *jomo* package in Rstudio [34].

Preliminary checks for associations between missingness and predictor values were conducted to check for obvious violations of the missing-at-random assumption. Imputations were assessed for consistency by comparing density plots, histograms, and summary statistics across imputations and back to the complete values, within and across studies. The model coefficients and predictive performance measures were then estimated in each imputed dataset separately, before being combined across imputations using Rubin’s Rules [35].

#### Model Development

Outcomes were modelled using multilevel mixed-effects regression with a random intercept, to account for potential heterogeneity in baseline risk of work absence, or mean presenteeism score, across the model development populations. Models were fitted using restricted maximum likelihood (REML), with an unstructured variance–covariance for the random effects [36]. Binary outcomes of work absence were modelled using multilevel logistic regression, while the continuous outcome of presenteeism was modelled using multilevel linear regression.

Continuous predictors of age, baseline pain intensity score and baseline work performance were modelled on their continuous scale. Pain duration at baseline was already categorised into clinically meaningful groups in each included study, thus exact values were not known. Cut points in pain duration were chosen to ensure consistent groupings across studies. Fractional polynomial terms up to the second order were tested for the continuous variables in a complete case analysis to determine the best functional form for each variable in the presence of all other predictors. All continuous variables were found to be best modelled linearly. No statistical selection of predictors took place [37].

Heuristic shrinkage was calculated following the method proposed by Van Houwelingen and le Cessie [38] in each imputation, and was pooled across imputations using Rubin’s rules to obtain the average shrinkage factor. This pooled shrinkage factor was then applied to each beta coefficient, and subsequently average intercept values were re-estimated (holding fixed the shrunken beta coefficients) to ensure predictions were correct on average.

#### Model Validation

An internal–external cross-validation (IECV) approach was used for model validation, as individual participant data (IPD) were available from multiple studies and to allow maximum data to be used for model development [39, 40]. This approach involved cross-validation methods, omitting one study in turn from the development data, developing a model and then validating it in the omitted study. This was repeated multiple times (cycles) until each study had been used for validation once. The full modelling procedure, as described in the previous section, was conducted in each cycle, including assessment of overfitting with model coefficients shrunken using a cycle-specific estimate of heuristic shrinkage. The apparent performance of the models was also calculated, in which the model was applied directly back to the development data, without any further adjustment for optimism in performance estimates.

Model performance was assessed using measures of calibration (calibration slope, calibration-in-the-large (CITL), and the ratio of Observed to Expected outcome proportions (O/E)) and discrimination (C-statistic). Model calibration in each IECV cycle was further visualised using calibration plots with smooth calibration curves overlaid.

Following IECV there were multiple values for each performance statistic (one from each cycle), which were then combined using a random effects meta-analysis to gain summary estimates of “external” model performance across all studies. Heterogeneity in model performance across IECV cycles is summarised using the ${\tau }^{2}$ statistic and visualised using forest plots.

All analyses were performed using Stata MP Version 16 (StataCorp) and R version 3.2.2. This paper adheres to the TRIPOD checklist for the transparent reporting of multivariable prediction models [41], see Appendix III.

## Results

### Study Population Characteristics

Across the six studies, five [24–28] were available for prediction of 6-month work absence (2179 participants, 215 absence events), three [26–28] for predicting 6-month presenteeism (1218 patients), and two [15, 26] for predicting 12-month work absence (408 patients, 132 absence events).

Patients were predominantly employed in professional or managerial roles in all studies, with proportions of patients in this category ranging from 56 to 71%. Distributions of job types were relatively consistent across studies, with around a quarter of each reporting they had manual occupations. Of those studies that measured presenteeism at baseline, three-quarters had a median reported score of 5 on a 0–10 scale, with lower and upper quartiles of 2 and 7, respectively. Three studies recorded whether patients reported taking work absence in the prior six months, with between 28% and 40% of patients confirming that they had taken time off due to their MSD. Patient characteristics for all studies are summarised in Table 2.

**Table 2:** Study population summary, detailing demographic information across individual study populations and in the total population used for the development of each prediction model

	Individual study populations						Modelling population totals		
	BEEP	KAPS	STEMS	SWAP	STarT MSK-pilot	STarT MSK-MT	6m absence	12m absence	6m presenteeism
*n*	214	747	432	338	232	554	2179	408	1218
Age, mean (SD)	56.4 (7.1)	49.6 (11.3)	49.6 (11.6)	48.7 (10.3)	51.2 (12.4)	50.2 (12.7)	50.6 (11.7)	50.5 (9.7)	52 (12.3)
Gender, female	105 (49)	430 (58)	230 (53)	195 (58)	140 (60)	313 (57)	1218 (56)	228 (56)	683 (56)
Multisite pain	199 (93)	336 (45)	330 (34)	219 (65)	18 (8)	45 (8)	704 (32)	200 (49)	169 (14)
Pain score (baseline), median (LQ-UQ)	4 (3–5.5)	5 (3–7)	7 (7–9)	7 (6–8)	6 (5–8)	7 (5–8)	6 (4–8)	6.3 (6–8.2)	6.3 (5–8)
Pain duration									
< 3 months	–	173 (23)	180 (42)	111 (33)	66 (28)	134 (24)	553 (25)	147 (36)	380 (31)
3–6 months	–	105 (14)	53 (12)	41 (12)	29 (13)	97 (18)	284 (13)	46 (11)	179 (15)
7–12 months	–	102 (14)	49 (11)	46 (14)	33 (14)	77 (14)	261 (12)	55 (13)	159 (13)
Over 1 year	147 (69)	367 (49)	132 (21)	131 (39)	104 (45)	241 (44)	1052 (48)	152 (37)	477 (39)
Job type									
Professional/managerial	141 (71)	476 (65)	256 (61)	188 (56)	357 (64)	160 (69)	1390 (64)	241 (59)	773 (64)
Intermediate	20 (10)	85 (12)	53 (13)	52 (15)	59 (11)	21 (9)	238 (11)	51 (13)	133 (11)
Manual	37 (19)	169 (23)	112 (27)	94 (28)	138 (25)	50 (22)	506 (23)	109 (27)	300 (25)
Comorbidities									
Diabetes	14 (7)	48 (6)	29 (7)	–	13 (6)	33 (6)	137 (6)	16 (4)	75 (6)
Respiratory problem	29 (14)	89 (12)	69 (16)	–	35 (15)	71 (13)	293 (13)	24 (6)	175 (14)
Heart problem	14 (7)	129 (17)	113 (26)	–	49 (21)	101 (18)	406 (19)	47 (12)	263 (22)
Anxiety/depression	46 (22)	123 (16)	125 (29)	105 (31)	43 (19)	136 (25)	473 (22)	141 (39)	304 (25)
Other	80 (37)	181 (24)	167 (39)	–	40 (17)	93 (17)	561 (26)	177 (43)	300 (25)
Time off in previous 1 month	–	–	128 (30)	–	–	–	128 (6)	57 (14)	128 (11)
Days off in previous 1 month*, median (LQ-UQ)	–	–	7 (2–14)	–	–	–	7 (2–14)	7 (2–14)	7 (2–14)
Time off in previous 6 months	–	300 (40)	–	–	66 (28)	177 (32)	543 (25)	–	371 (30)
Days off in previous 6 months*, median (LQ-UQ)	–	10 (4–21)	–	–	6.5 (3–21)	8 (3–21)	8 (3–21)	–	7 (3–21)
Presenteeism at work, median (LQ-UQ)	–	–	5 (2–7)	6 (5–9)	5 (2–7)	5 (2–7)	5 (2–7)	6 (3–8)	5 (2–7)
Outcomes recorded at follow-up									
Any absence at 6 months	17 (8)	21 (3)	41 (9)	–	41 (18)	95 (17)	215 (10)	–	–
Presenteeism at 6 months, median (LQ-UQ)	–	–	4 (2–6)	–	3 (1–6)	3 (1–6)	–	3 (1–6)	–
Any absence at 12 months	–	–	34 (17)	98 (46)	–	–	–	–	132 (32)

Numbers are *n* (%) unless otherwise stated

*SD* standard deviation, *LQ-UQ* lower quartile to upper quartile

– Indicates that data were not collected on this variable at this time point

^*^Number of days summarised only in those reporting that they had taken time off within the time frame

### Predicting Work Absence at Six Months

Conditional on other variables in the model, the strongest predictor of any work absence over the 6-month follow-up period was having taken any absence in the prior 6 months, with an increase in odds for those with a prior absence. Being female, having multisite MSDs, and being in intermediate or manual job types reduced an individual’s odds of absence, while both higher baseline pain intensity and higher baseline presenteeism increased the odds of absence for each unit increase in score. All model predictors and associated parameters are shown in Table 3.

**Table 3:** Prognostic model details (coefficients, constant terms with variance, and shrinkage estimates) for models to predict any absence over 6 months and 12 months, and presenteeism at 6 months

Variable	Any absence at 6 months: coefficients	Presenteeism at 6 months: coefficients	Any absence at 12 months: coefficients
Age*	−0.016	−0.016	−0.005
Female	−0.307	−0.124	0.003
Multisite pain	−0.141	−0.028	0.172
Baseline pain score*	0.127	0.198	0.101
Pain duration			
< 3 months	Reference	Reference	Reference
3–6 months	0.308	0.067	−0.76
7–12 months	0.008	0.186	−0.308
> 12 months	0.432	0.722	0.061
Job type			
Professional/managerial	Reference	Reference	Reference
Intermediate	−0.015	0.212	0.534
Manual	−0.315	0.387	0.452
Anxiety/depression	0.370	0.317	0.457
Comorbidity (yes/no)	−0.024	0.424	–
Work absence in previous 6 months	1.465	0.252	–
Presenteeism at work*	0.250	0.375	0.129
Constant**	−3.493	0.309	−2.533
Random effect**, sd(constant)	0.794	0.118	0.613
Shrinkage	0.9464	0.9746	0.7832

^*^Coefficients refer to the effect for a one-unit increase in the variable e.g., per one-year increase in age

^**^ Constant and random effects terms were re-estimated after adjustment for optimism to maintain overall model calibration

Upon IECV validation, the model showed good calibration on average across all studies with a pooled calibration slope of 0.93 (95% CI: 0.41–1.46, ${\tau }^{2}$= 0.123), although calibration performance did vary considerably across cycles (see Table 4, and supplementary figures S4a and S5a). In particular, poor calibration was seen when the model was applied in the KAPS cohort study alone, with substantial overestimation of absence risk, on average. The C-statistic was more consistent across cycles, with a pooled value of 0.76 (95% CI: 0.66–0.86, ${\tau }^{2}$= 0.004) suggesting that the model on average discriminated well between those who went on to take any work absence and those who did not, even where calibration was sub-optimal.

**Table 4:**
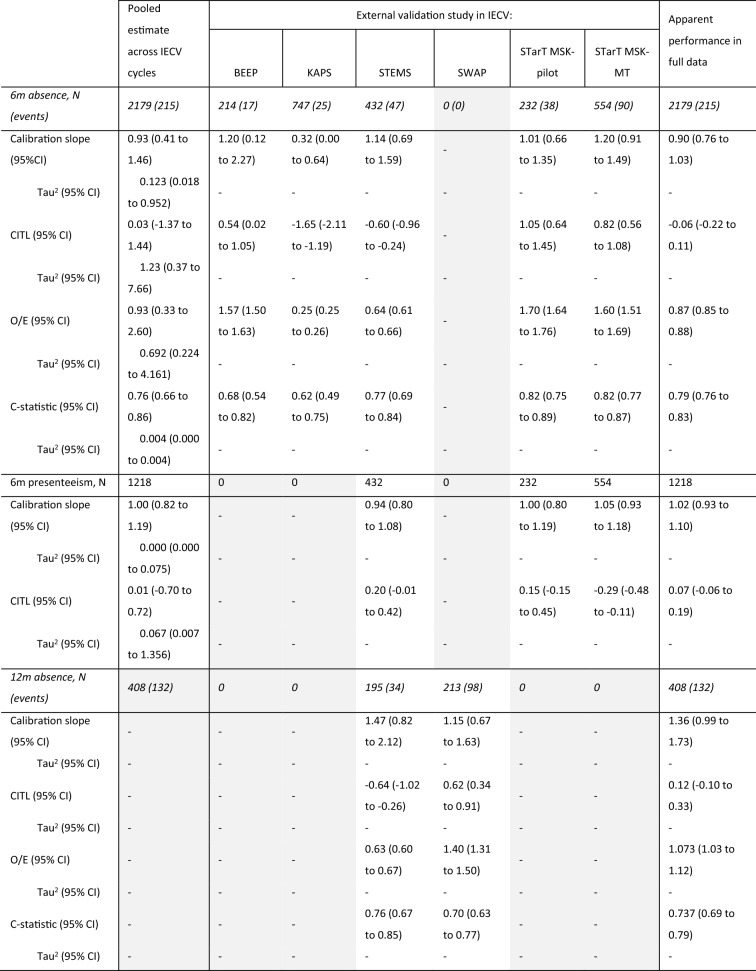
Predictive performance of the 6 month absence and presenteeism models with average intercept in each IECV cycle: the external validation performance in each study, for the cycle in which it was excluded from model development, with pooled effect estimates across studies, and apparent performance in the full data, without accounting for clustering of data by study

Grey boxes indicate studies that did not contribute to the analysis for that model

Numbers reported for the 12-month absence model are apparent model performance in study subgroups

*6m* 6-months, *12m* 12 months, *CI* confidence interval, *CITL* calibration-in-the-large, *O/E* observed /expected ratio

### Predicting Presenteeism at Six Months

Taking account of other variables in the model, the strongest association with 6-month presenteeism was having comorbidities and having a pain duration of more than 12 months at consultation, with an increase in presenteeism score for those with other health conditions and longer pain durations.

**Fig. 1:**
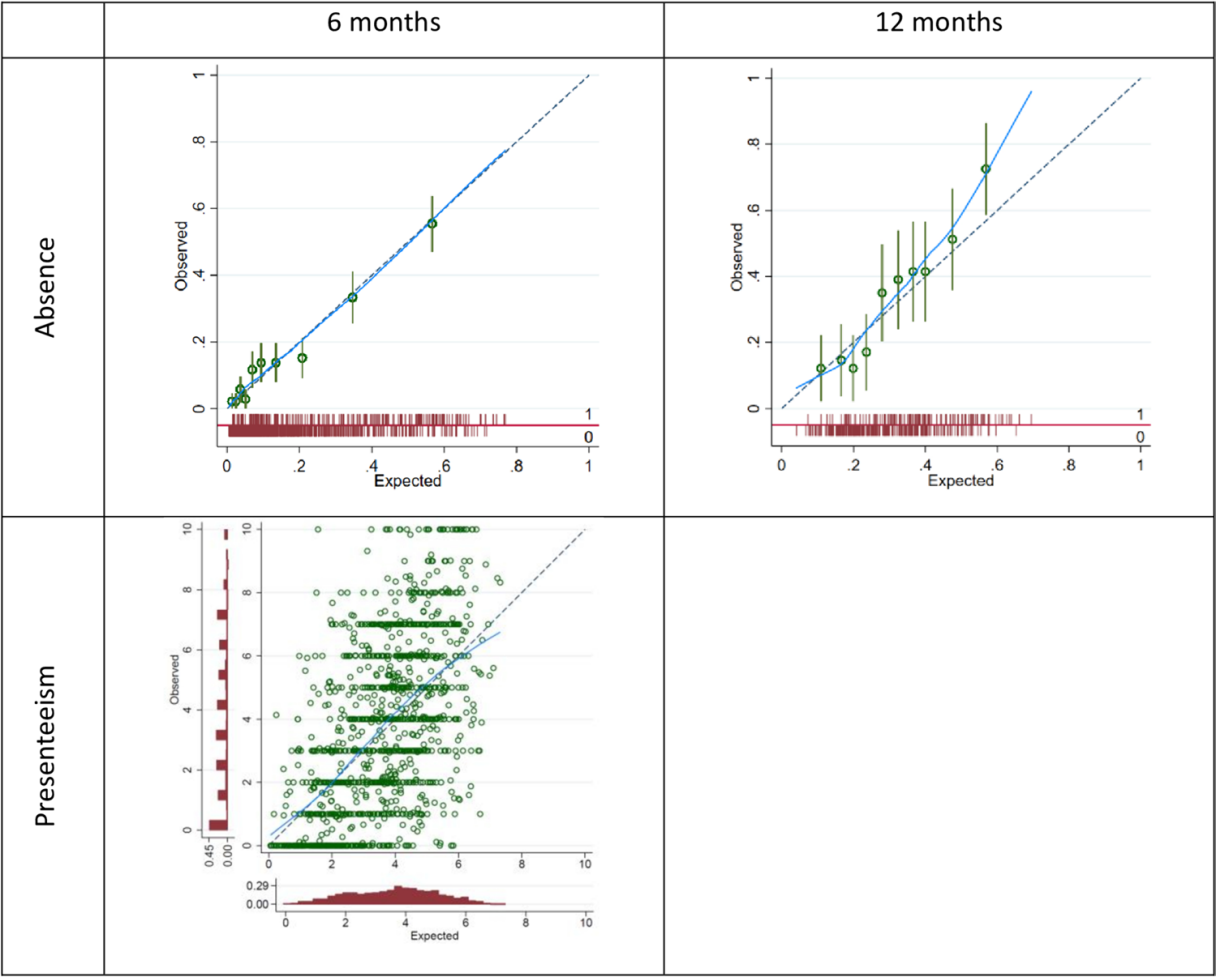
Calibration plots for models to predict six-month absence, six-month presenteeism, and 12-month absence. Each plot shows the performance of the final shrunken model, when applied across all studies combined (without accounting for clustering of data by study)

Calibration performance of the model was near-perfect on average, with a pooled calibration slope of 1.00 (95% CI: 0.82–1.19, ${\tau }^{2}\le$ 0.001) and pooled CITL of 0.01 (96%CI: −0.70 to 0.72, ${\tau }^{2}$= 0.067) across studies. Although calibration was very good on average, on the individual level predicted presenteeism scores can be seen to vary substantially from observed outcomes (see Fig. 1 and supplementary figures S1b and S5a), meaning predictions for individuals may be unreliable.

### Predicting Work Absence at 12 Months

Given only two studies contributed to the analyses regarding 12-month absence, the IECV approach was deemed inappropriate and so only apparent performance of this model was assessed. The model can be seen to be underfit to the data, a consequence of shrinkage due to the substantial estimate of overfitting in the initial model (heuristic shrinkage = 0.7832). Despite miscalibration in the full data and in subgroups, the model’s discrimination performance was adequate, with estimates of 0.76 (95% CI: 0.67–0.85) and 0.70 (95% CI: 0.63–0.77) in the STEMS and SWAP subgroups respectively (see Table 1 for study descriptions). After adjusting for this overfitting, the model showed reasonable discrimination across both studies, with a C-statistic of 0.737 (95% CI: 0.69–0.79).

## Discussion

### Summary of Main Findings

We have developed and validated prediction models, based on a combination of a patient’s demographic details, health status and job-type, to estimate an individual’s risk of any work absence over 6 and 12 months, and their expected level of presenteeism over 6 months, following consultation in primary care for MSDs. A total of 10 predictor variables were included for predictions of 6-month absence and 6-month presenteeism, including patient demographics (age, sex, occupational class [(i) managerial/administrative/professional; (ii) intermediate; (iii) routine and manual]), pain features (multisite or single site pain, pain intensity at baseline, pain duration), comorbidities (anxiety/depression, any other comorbidity), work absence in previous six months and current performance at work. For prediction of 12-month absence, predictors regarding previous absence and comorbidities were not consistently recorded across studies, leaving just eight available predictors. The 6-month absence and presenteeism models may be implemented during a consultation with a primary care clinician, to help inform targeted interventions designed to reduce the impact of MSDs on work absence and productivity. However, the 12-month absence model would need further work to be able to confidently inform clinical practice, due to the limited data available for its derivation and resulting miscalibration on validation.

On IECV, the 6-month absence model was well calibrated on average, with a pooled calibration slope of 0.93 (95% CI: 0.41–1.46, ${\tau }^{2}$= 0.123) and a CITL of 0.03 (95% CI: (−1.37 to 1.44), ${\tau }^{2}$= 1.23), although substantial heterogeneity in calibration performance was seen across studies. In particular, considerable overestimation of absence risk was seen when the model was applied in the KAPS study alone. This is possibly due to the lower prevalence of 6-month absence in the KAPS population, with only 3% (21 individuals) with an absence over 6 months following consultation. The model’s discrimination performance was more consistent, however, with a pooled C-statistic of 0.76 (95% CI: 0.66–0.86, ${\tau }^{2}$= 0.004). The results across IECV cycles suggest the model can separate well between those who went on to take work absence and those who did not across populations, despite some variation in model calibration (see Figure S2a).

Calibration performance of the model to predict continuous presenteeism at six months was good on average, with a pooled calibration slope and pooled CITL of 1.00 (95% CI: 0.82–1.19, ${\tau }^{2}\le$ 0.001) and 0.01 (96%CI: −0.70 to 0.72, ${\tau }^{2}$= 0.067) respectively. While this model predicted well on average, across the whole population, it is worth noting that there was still considerable variation evident between the predicted and observed presenteeism scores on the individual level.

Full IECV was not possible for the 12-month absence model, as only two studies were available with the outcome measured at this time point. On internal validation, the model showed a large amount of overfitting to the model development data (heuristic shrinkage = 0.7832). After adjusting for this overfitting, the model showed reasonable discrimination across both studies, with a C-statistic of 0.737 (95% CI: 0.69–0.79), although it was miscalibrated at the extremes, with evidence of underestimation of risk in those at higher risks.

### Strengths and Limitations of this Work

An important strength of this study was the sample size available for development of most models. For the primary objective (to model any work absence over 6 months of follow-up), the model development sample was sufficiently large to meet current minimum sample size recommendations [30], while incorporating the desired clinically important predictors, identified through the literature (PROSPERO CRD42020219452) and through expert clinical opinion. Similarly, there was more than enough data to meet the recommendations for the secondary analysis of modelling presenteeism at six months, while including all chosen predictors [31].

Unfortunately, insufficient data were present in the available studies to meet the recommended minimum sample size for the development of a model to predict 12-month absence. These results should be viewed with caution with further external validation, as it is possible the model would not perform well in new individuals.

Model development for all outcomes involved using mixed populations through multiple studies, all with similar recruitment regions and inclusion criteria, methodologies included one cohort study, RCTs and cluster RCTs which means the influence of treatments should be minimised due to randomisation (see Tables 1 and 2 for study descriptions). The generalisability of RCT populations can be limited, however the trials included in this analysis all used broad inclusion criteria meaning the design is unlikely to greatly affect generalisability of the models. This meant that we were able to test model generalisability across similar populations through use of IECV, whilst still allowing maximum data to be used for model development. Although model generalisability has been tested there was variability in calibration, further external validation in populations of different compositions e.g., different healthcare settings, or those with specific MSDs will be important, to provide information regarding the model’s transportability.

One further limitation to generalisability is that the populations included in these studies contained disproportionately high numbers of people in the higher socioeconomic status banding, compared to the population in North Staffordshire where the studies were based. Thus, it is possible that the models may not perform as well in those with a lower socioeconomic status, as they were underrepresented at model development.

While there are strengths in terms of participant numbers, there were some drawbacks to having developed these models across multiple previous studies, primarily that we were restricted to the use of predictors that were consistently collected. While some important predictors were able to be accommodated through multiple imputation of systematically missing information [32, 33], it is possible that some important and strong predictors of work outcomes may have been omitted from model development due to them not having been collected or recorded consistently across enough of the included studies. For example, for the model predicting 12-month work absence, previous work absences were not recorded at consistent time-points across the studies used for model development and so this predictor could not be included, despite being the strongest predictor of absence within 6 months.

All predictors included in these models were self-report measures, but omit any information arising from the objective patient assessment. This decision was made due to a lack of a standardised assessment items for patients consulting in primary care with MSD, and the availability of self-report items being more consistent across our model development studies. In practice, primary care clinicians assess MSD patients in varied ways and our use of self-report predictors could enable consistent application of models, despite wide variation in objective assessments. Indeed, previous work suggests that clinical examination and imaging results add little to predictions in patients with low back pain, over predictors such as age, pain features, or depression, all of which we included in our models [42, 43]. Predictor variables were reported 1–4 weeks after consultation due to recruitment methods. While the values of many of the included predictors would not be expected to vary over short periods of time, some (e.g., current pain intensity) would. It is recommended that prognostic variables are collected at consultation as this is the point at which decisions about interventions are most commonly made, therefore testing these models at the point of consultation is a key next step to assess their usefulness in practice and to guide timing of their use [44, 45].

Regarding the prediction of 12-month absence, model development was conducted in the limited population of patients who reported they were still working one year after consultation; thus, predictions can only be assumed to apply to an individual if they were to still be in work at that time. While ensuring the population was clearly defined, this restriction resulted in our model missing those who were no longer working but had nevertheless taken an absence during the 12-month period where they had still been at work. This omits the higher risk, and arguably more relevant population, of people who stop work within the year due to their MSD.

### Comparison with Previous Literature

Predicting work absence in patients with MSD is challenging, with other studies in this field finding that there are many and varied predictors, often with inconsistent measurement of predictor variables between studies [46–48]. A number of models have been developed that also predict work outcomes, the most commonly used is the Orebro Musculoskeletal Pain Questionnaire [21, 49, 50]. The full version of this questionnaire contains 25 questions (of which 21 are scored) and it has good predictive ability with a sensitivity of 89% and specificity of 65% for absenteeism. The short-form version of the Orebro questionnaire includes 10 questions and has been demonstrated to be useful as an early screening tool in primary care with a sensitivity of 0.75 and specificity of 0.78 [22]. We were unfortunately unable to validate either form of the Orebro questionnaire in our populations, as necessary predictor items had not been measured, thus we were unable to directly compare the performance of our new models with that of Orebro.

Whilst there are some similarities in the measures included in Orebro (e.g. pain intensity, anxiety/depression), there are also some differences in particular the populations in which each model was developed, with Orebro developed in a population with back pain only. Whilst the Orebro has been used in patients with other regional pain symptoms, specifically neck and shoulder pain, its predictive ability with broader MSDs and multisite pain is not clear. Other models also demonstrate reasonable predictive ability but again these are lengthy and do not always include previous work absence [20, 51, 52]. Furthermore, none of these models predict presenteeism, which may have a bigger impact on work than absenteeism [23]. However, in line with our findings, other research has consistently identified previous sickness absence as being strongly associated with future absence, indicating that this variable is an important predictor of future work absence [51, 53–56]. The Orebro questionnaires do not include previous work absence but do include a measure of “work expectation” in 3 months, and various measures of self-perceived/rated work ability have been demonstrated to predict absence over time [54].

### Implications for Policy and Practice

By understanding which patients are at higher risk of work absence, preventative consultations (or targeted vocational advice and support interventions/referral to occupational health services) may be put in place and support the appropriate use of scarce vocational resources [57]. Discrimination performance of our work absence models is consistent with that of previous models to predict work absence in more general populations [54, 58], with the benefit of being tailored specifically to the MSD population. Identification of patients at risk of work absence may be useful for targeting work-focused interventions and occupational health strategies, such models could be valuable when used as a part of a wider assessment of patients with MSD to ensure appropriate support is offered.

To our knowledge this is the first time presenteeism has been predicted, it is more commonly included as a predictor variable in prognostic models of absence [59] or has been predicted using a very narrow prognostic model focusing on one concept rather than including a wider range of prognostic factors [60]. Predicting presenteeism may be useful to identify those patients who are likely to struggle with their MSD at work, allowing the patient and clinician to plan mitigation strategies to decrease presenteeism, for example ensuring appropriate support networks are in place, consideration of frequency and timing of breaks or planning work tasks to better manage pain.

Future research should focus on a comparison between our model and the Orebro questionnaires in addition to further validation of the 6-month absence and presenteeism models with other MSD populations in other settings, to ensure the models sufficiently and accurately predict work absence and performance across settings. Additionally, these models would need to be tested in clinical practice to explore their usability and feasibility in the practice setting, prior to considering wider implementation. The impact of the proposed models could then be considered in terms of potential for targeting interventions, and effects on patient and cost outcomes.

## Conclusion

The developed models predict 6-month work absence risk and presenteeism score with sufficient accuracy, on average, in adults consulting with MSD. This information could be useful to help support shared decision-making and to target occupational health interventions at those who are at risk of work absence over 6 months. Further validation of these models is needed in other populations, to confirm predictive performance, additionally testing in clinical practice to explore usability and feasibility is required before their use can be recommended or their impact for patient care can be fully assessed.

## Supplementary Information

Below is the link to the electronic supplementary material.

Supplementary file1 (DOCX 3075 kb)
