# Supplementary material for: Musculoskeletal Health and Work: Development and Internal–External Cross-Validation of a Model to Predict Risk of Work Absence and Presenteeism in People Seeking Primary Healthcare
Source: J Occup Rehabil. 2024 Jul 4;35(3):578–91. doi: 10.1007/s10926-024-10223-w (PMC12360984; doi:10.1007/s10926-024-10223-w)
Supplement: Supplementary file 1 — Supplementary file1 (DOCX 3075 kb) [file 10926_2024_10223_MOESM1_ESM.docx]

Supplementary materials: Musculoskeletal health and work: development and internal-external cross-validation of a model to predict risk of work absence and presenteeism in primary care consulters

Lucinda Archer (0000-0003-2504-2613)^1,2,3^, George Peat^1,4^, Kym IE Snell (0000-0001-9373-6591)^2,3^, Jonathan C Hill^1^, Kate M Dunn^1^, Nadine E Foster^1,5^, Annette Bishop^1^, Danielle van der Windt^1^, Gwenllian Wynne-Jones^1*^

^1^ School of Medicine, Keele University, Staffordshire, ST5 5BG, United Kingdom

^2^ Institute of Applied Health Research, University of Birmingham, Edgbaston, Birmingham, B15 2TT

^3^ National Institute for Health and Care Research (NIHR) Birmingham Biomedical Research Centre, UK

^4^ Centre for Applied Health and Social Care, Sheffield Hallam University, Sheffield, S10 2BP, United Kingdom

^5^ Surgical Treatment and Rehabilitation Service (STARS) Education and Research Alliance, The University of Queensland and Metro North Hospital and Health Service, Queensland, Australia.

* corresponding author: Gwenllian Wynne-Jones, email: [g.wynne-jones@keele.ac.uk](mailto:g.wynne-jones@keele.ac.uk)

# Other information

## Funding

This paper presents work conducted as part of the Centre for Musculoskeletal Health and Work funded by the Medical Research Council and Versus Arthritis. LA and KIES are supported by funding from the NIHR Birmingham Biomedical Research Centre at the University Hospitals Birmingham NHS Foundation Trust and the University of Birmingham. The views expressed are those of the author(s) and not necessarily those of the NHS, the NIHR or the Department of Health and Social Care.

The funding bodies had no role in the design of the study and collection, analysis, interpretation of data, or in writing the manuscript.

## Acknowledgements

The authors would like to thank the patients who gave up their time to participate in the studies from which our data came and the participating GP Practices.

Table of Contents

[Other information 1](#_Toc165295631)

[Funding 1](#_Toc165295632)

[Acknowledgements 1](#_Toc165295633)

[Appendix I: extended methods 4](#_Toc165295634)

[Outcome definitions 4](#_Toc165295635)

[6-month absence 4](#_Toc165295636)

[6-month presenteeism 4](#_Toc165295637)

[12-month absence 5](#_Toc165295638)

[Predictor measurement definitions across studies 6](#_Toc165295639)

[Sample size 8](#_Toc165295640)

[6-month absence 8](#_Toc165295641)

[6-month presenteeism 8](#_Toc165295642)

[12-month absence 8](#_Toc165295643)

[Missing data 8](#_Toc165295644)

[Decision curve analysis 10](#_Toc165295645)

[Appendix II: supplementary tables and figures 11](#_Toc165295646)

[Table S1: Table of missingness in predictor measurements across datasets. “-“ denotes no missing values. 11](#_Toc165295647)

[Table SX: Prognostic model details (coefficients, constant terms with variance) before shrinkage, with 95% confidence intervals 12](#_Toc165295648)

[Table S2: Prognostic model details (odds ratios, constant terms with variance, and shrinkage estimates) for models to predict any absence by 6 months, after adjustment for overfitting, for each IECV cycle. 13](#_Toc165295649)

[Figure S1a: Apparent calibration of 6-month absence model (after adjustment for overfitting), by study subgroups, and on average across all data 14](#_Toc165295650)

[Figure S1b: Apparent calibration of 6-month presenteeism model (after adjustment for overfitting), by study subgroups, and on average across all data 15](#_Toc165295651)

[Figure S1c: Apparent calibration of 12-month absence model (after adjustment for overfitting), by study subgroups, and on average across all data 16](#_Toc165295652)

[Figure S2a: Distribution of predicted probabilities for absence at 6 months (by outcome status) for each study in the IECV cycle in which it was excluded from model development, and of the final shrunken model in all datasets combined 17](#_Toc165295653)

[Figure S2b: Distribution of predicted values for presenteeism at 6 months for each study in the IECV cycle in which it was excluded from model development, and of the final shrunken model in all datasets combined 18](#_Toc165295654)

[Figure S2c: Distribution of predicted values for probability of absence at 12 months of the final shrunken model, for each study subgroup and in both datasets combined 19](#_Toc165295655)

[Figure S3a: Decision curves for models to predict 6 and 12-month absence. Each plot shows the performance of the final shrunken model, when applied across all studies combined (without accounting for clustering of data by study). Clinically relevant risk threshold range, defined *a priori* based on clinical expert opinion and previous literature as being decision thresholds anywhere between 0.26 and 0.49, is highlighted. 20](#_Toc165295656)

[Figure S3b: Decision curves for model to predict the probability of absence at 6 months for each study in the IECV cycle in which it was excluded from model development, and of the final shrunken model in all datasets combined. Clinically relevant risk threshold range between 0.25 and 0.5 22](#_Toc165295657)

[Figure S3c: Decision curves for model to predict the probability of absence at 12 months of the final shrunken model, for each study subgroup and in both datasets combined. Clinically relevant risk threshold range between 0.25 and 0.5. 23](#_Toc165295658)

[Figure S4a: Forest plots of pooled model performance estimates for the 6-month absence model, across IECV cycles 24](#_Toc165295659)

[Figure S4b: Forest plots of pooled model calibration estimates for the 6-month presenteeism model, across IECV cycles 25](#_Toc165295660)

[Figure S5a: External calibration of 6-month absence model (after adjustment for overfitting), for each study in the IECV cycle in which it was excluded from model development 26](#_Toc165295661)

[Figure S5b: External calibration of 6-month presenteeism model (after adjustment for overfitting), for each study in the IECV cycle in which it was excluded from model development 27](#_Toc165295662)

[References 29](#_Toc165295663)

# Appendix I: extended methods

## Outcome definitions

### 6-month absence

| Study | Outcome measurement method |
| --- | --- |
| BEEP | Have you taken time off work during the last 6 months because of your knee problem, including time off to visit any health care professional? |
| KAPS | Have you taken time off work in the last 6 months because of your pain problem? |
| STEMS | Have you taken time off work during the last 6 months because of your pain problem? (Please tick one box only) |
| STarT MSK-MT | Have you taken time off work in the last 6 months because of your [location] pain? |
| STarT MSK-pilot | Have you taken time off work in the last 6 months because of your pain problem? |

### 6-month presenteeism

| Study | Outcome measurement method |
| --- | --- |
| STEMS | “On average to what extent has your pain or related problem affected your performance at work over the past 6 months? (Please tick one box only)”, where zero indicated “Not at all” and 10 indicated “So bad I am unable to do my job”. |
| STarT MSK-MT | “On average to what extent has your pain or related problem affected your performance at work over the past 6 months? (Please tick one box only)”, where zero indicated “Not at all” and 10 indicated “So bad I am unable to do my job”. |
| STarT MSK-pilot | “On average to what extent has your pain or related problem affected your performance at work over the past 6 months? (Please tick one box only)”, where zero indicated “Not at all” and 10 indicated “So bad I am unable to do my job”. |

### 12-month absence

| Study | Outcome measurement method |
| --- | --- |
| STEMS | Have you taken time off work during the last 6 months because of your pain problem? (Please tick one box only) |
| SWAP | Sometimes when people are off work sick they “self-certify”, this means they are off work for only a few days and do not need a note from their doctor.  In the last 8 months have you self-certified time off work because of your pain?  In the last 8 months (since your previous questionnaire) have you been given any “Sick Notes” or “Fit Notes” from your doctor because of your pain? |

## Predictor measurement definitions across studies

|  | **Measurement method/definition in study:** | | | | | |
| --- | --- | --- | --- | --- | --- | --- |
| **Predictor** | **BEEP** | **KAPS** | **STEMS** | **STarT MSK-MT** | **STarT MSK-pilot** | **SWAP** |
| Age | Date of birth | Date of birth | Date of birth | Date of birth | Date of birth | Date of birth |
| Sex | Female/Male | Female/Male | Female/Male | Female/Male | Female/Male | Female/Male |
| Multisite pain | Body manikin | Presence of widespread pain: yes/no  Body manikin | Body manikin | Widespread pain | Widespread pain | Body manikin |
| Baseline pain score | Visual analogue scale 0-10 | Numerical rating scale 0-10 | How much bodily pain have you had in the past week?*  None  Very mild  Mild  Moderate  Severe  Very severe | Pain intensity (at point of GP consultation): Numerical rating scale 0-10 | Pain intensity (at point of GP consultation): Numerical rating scale 0-10 | In the last 2 weeks how intense was your usual pain? Numerical rating scale 0-10 |
| Pain duration | When did you first start having this problem with your knee?  More than 1 year but less than 5 years More than 5 years but less than 10 years I have had this knee problem for more than 10 years | Time since last whole month pain free: <3 months 3-6 months 7-12 months 1-2 years 3-5 years 6-10 years >10 years | Duration of current episode: Less than 2 weeks 2-6 weeks 6-12 weeks 3-6 months 7-12 months More than 12 months | Time since last whole month pain free: <3 months 3-6 months 7-12 months 1-2 years 3-5 years 6-10 years >10 years | Time since last whole month pain free: <3 months 3-6 months 7-12 months 1-2 years 3-5 years 6-10 years >10 years | Duration of current episode: Less than 2 weeks 2-6 weeks 6-12 weeks 3-6 months 7-12 months More than 12 months |
| Job type** | Current/most recent job title | Current/most recent job title | Current/most recent job title | Current/most recent job title | Current/most recent job title | Current/most recent job title |
| Anxiety / depression | PHQ 8 GAD 7 | SF36 mental component | SF36 mental component | Distress numerical rating scale 0 - 10 | Distress numerical rating scale 0 - 10 | HADs |
| Other comorbidity | Have you ever been told you have any of the following? High blood pressure; angina; heart failure; stroke; depression; osteoporosis; diabetes; asthma; bronchitis; heart attack | Self-reported diagnosed comorbidities | High blood pressure; heart problems; stroke; diabetes; depression or anxiety; osteoporosis; arthritis; asthma or bronchitis; liver disease; cancer; other | Self-reported diagnosed comorbidities | Self-reported diagnosed comorbidities | [Not recorded] |
| Work absence in the previous 6 months | How many days/weeks or months were you absent from work due to your knee problem | Number of days absence from work in previous 6 months | Number of days absence from work in previous 1 month | Time off work in the last 6 months yes/no | Time off work in the last 6 months yes/no | [Not recorded] |
| Presenteeism at work | [Not recorded] | [Not recorded] | Single item question impact of pain productivity at work numerical rating scale: 0-10 | Single item question impact of pain productivity at work numerical rating scale: 0-10 | Single item question impact of pain productivity at work numerical rating scale: 0-10 | Single item question impact of pain productivity at work numerical rating scale: 0-10 |

* Standardised to a 0-10 scale for consistency across studies. ** Allocated to “Professional/managerial”, “Intermediate”, and “manual” (National Statistics Socio-economic Classification operational categories, three class) by the study authors

## Sample size

### 6-month absence

The sample size for model development was fixed at a total of 2179 participants (215 events) due to the size of the available datasets. Riley et al 2019 (1) criteria for the development of a prediction model with a binary outcome suggest a sufficient sample was available across the five model development datasets to include a maximum of 16 predictor parameters at model development, based on an expected Cox-Snell R-squared of 0.067 (Nagelkerke’s R-squared of 0.14 (2)) and an outcome prevalence for work absence by 6 months of 10%, while targeting shrinkage estimate of at most 0.9. Thus, our data was sufficient to meet the recommended minimum requirements to build our intended model with 13 predictor parameters.

### 6-month presenteeism

The sample size for model development was fixed at a total of 1218 participants due to the size of the available datasets. Riley et al 2019 (3) criteria for the development of a prediction model with a continuous outcome suggest a sufficient sample was available across the three model development datasets to include a maximum of 24 predictor parameters at model development, based on an expected R-squared of 0.15 (as suggest by Riley et al), and an expected average outcome value of 3.6 with standard deviation of 2.8. Thus, our data was sufficient to meet the recommended minimum requirements to build our intended model with 13 predictor parameters.

### 12-month absence

The sample size for model development was fixed at a total of 408 participants (132 events) due to the size of the available datasets. Riley et al 2019 (1) criteria for the development of a prediction model with a binary outcome suggest a sufficient sample was available across the two model development datasets to include a maximum of only 4 predictor parameters at model development, based on an expected Cox-Snell R-squared of 0.10 (Nagelkerke’s R-squared of 0.14 (2)), shrinkage of at most 0.9, and an outcome prevalence for work absence by 12 months of 33%. Thus, our data was insufficient to meet minimum recommended sample size criteria for this outcome, and the results of our modelling with 11 predictor parameters should be considered with this caveat.

## Missing data

Multiple imputation of the multilevel data was implemented using joint modelling, to impute missing data across datasets, retaining heterogeneity between and allowing for the clustering within the included datasets to maintain congeniality with the subsequent IPD meta-analysis model (4, 5). This further allowed for the imputation of two predictor variables that were systematically missing from two of the datasets (a summary of missing data is given in supplementary table S1). This approach was implemented using the *jomo* package in R studio (6).

One hundred imputed datasets were generated using joint modelling imputation, clustering by study, with a burn-in of 1000 imputations to stabilise and 100 iterations between successive imputations, considering random study-specific covariance matrices. The number of imputations was chosen to exceed the largest proportion of incomplete observations for all datasets, under the assumption that data were missing at random (7, 8). Preliminary checks for associations between missingness and predictor values were conducted to check for obvious violations of the missing-at-random assumption. Complete predictors were also included in the imputation models as auxiliary variables. The imputation model included all predictor and outcome variables.

The above process was repeated separated for the imputation of missing information for the 12-month absence model, with no imputation of systematically missing predictors (i.e., predictors were required to be at most sporadically missing from either dataset).

Imputations were assessed for consistency by comparing density plots, histograms, and summary statistics across imputations and back to the complete values. The model coefficients and predictive performance measures were then estimated in each imputed dataset separately, before being combined across imputations using Rubin’s Rules (9).

## Decision curve analysis

Clinical utility was assessed using net benefit analysis, where the benefits of using a work absence prediction model to make treatment decisions were offset against potential harms, across the full range of possible threshold probabilities. Threshold probabilities refer to potential cut-points in outcome risk values, that could be used to define those at “high risk” of work absence and so separate those who would receive additional support from those who would get usual care (10). Threshold probabilities of particular interest were defined *a priori*, based on clinical expert opinion and previous literature (11). These were defined as cut-points in work absence probabilities between 26% and 49%, thus a net benefit when using the model with a treatment decision threshold in this range was most desirable. The net benefit of using the model to guide decision-making was compared to model-blind methods of “treat none” (usual care for everyone) and “treat all” (a vocational advice and support intervention for all), and was visualised using decision curves.

The results of these analyses are reported in supplementary Appendix II, in figures S3a to S3c.

# Appendix II: supplementary tables and figures

## Table S1: Table of missingness in predictor measurements across datasets. “-“ denotes no missing values.

|  | BEEP  (n=214) | KAPS  (n=747) | STEMS  (n=432) | SWAP  (n=338) | STarT MSK-MT  (n=554) | STarT MSK-pilot  (n=232) |
| --- | --- | --- | --- | --- | --- | --- |
| Age | - | - | - | - | - | - |
| Female | - | - | - | - | - | - |
| Multisite pain | - | - | - | - | - | - |
| Baseline pain intensity score | 3 (1) | 3 (<1) | 1 (<1) | 4 (<1) | - | 1 (<1) |
| Pain duration | - | 6 (1) | 18 (4) | 9 (3) | 5 (2) | - |
| Job type | 16 (7) | 17 (2) | 11 (3) | 4 (<1) | - | 1 (<1) |
| Anxiety/depression | - | - | - | 2 (<1) | - | - |
| Other comorbidity | - | 2 (<1) | 4 (1) | 338 (100) | - | - |
| Work absence in previous 6 months | 214 (100) | 3 (<1) | 304 (70) | 338 (100) | 19 (8) | 1 (<1) |
| Work performance at baseline | 214 (100) | 747 (100) | 52 (12) | 3 (<1) | 6 (3) | 2 (<1) |
| Missing any predictor | 214 (100) | 747 (100) | 328 (76) | 338 (100) | 28 (5) | 5 (2) |
|  |  |  |  |  |  |  |
| 6 months: work absence | 56 (26) | 325 (44) | 195 (45) | 338 (100) | 169 (73) | 52 (9) |
| 12 months: work absence | 214 (100) | 747 (100) | 1 (<1*) | 7 (3*) | 232 (100) | 554 (100) |
| 6 months: work performance | 214 (100) | 747 (100) | 198 (46) | 338 (100) | 168 (72) | 53 (10) |

* N (%) missing from the working population at 12 months, STEMS – n=195, SWAP – n=213.

## Table SX: Prognostic model details (coefficients, constant terms with variance) before shrinkage, with 95% confidence intervals

| Variable | Any absence at 6 months:  coefficients (95% CI) | Presenteeism at 6 months:  coefficients (95% CI) | Any absence at 12 months:  coefficients (95% CI) |
| --- | --- | --- | --- |
| Age* | -0.016 (-0.033 to 0) | -0.017 (-0.031 to -0.002) | -0.007 (-0.033 to 0.019) |
| Female | -0.326 (-0.688 to 0.037) | -0.127 (-0.459 to 0.205) | 0.004 (-0.508 to 0.515) |
| Multisite pain | -0.15 (-0.652 to 0.352) | -0.028 (-0.531 to 0.474) | 0.219 (-0.292 to 0.73) |
| Baseline pain score* | 0.135 (0.043 to 0.227) | 0.203 (0.116 to 0.29) | 0.129 (-0.015 to 0.273) |
| Pain duration |  |  |  |
| <3 months | Reference | Reference | Reference |
| 3-6 months | 0.327 (-0.283 to 0.938) | 0.068 (-0.412 to 0.549) | -0.97 (-1.892 to -0.048) |
| 7-12 months | 0.008 (-0.664 to 0.68) | 0.191 (-0.346 to 0.728) | -0.393 (-1.162 to 0.375) |
| >12 months | 0.458 (0.01 to 0.905) | 0.74 (0.36 to 1.121) | 0.077 (-0.478 to 0.633) |
| Job type |  |  |  |
| Professional/managerial | Reference | Reference | Reference |
| Intermediate | -0.015 (-0.578 to 0.547) | 0.218 (-0.287 to 0.722) | 0.681 (-0.027 to 1.39) |
| Manual | -0.334 (-0.767 to 0.099) | 0.397 (0.011 to 0.782) | 0.576 (0.005 to 1.148) |
| Anxiety/depression | 0.392 (-0.003 to 0.786) | 0.325 (-0.051 to 0.702) | 0.583 (0.043 to 1.124) |
| Other comorbidity | -0.026 (-0.439 to 0.388) | 0.435 (0.09 to 0.78) | - |
| Work absence in previous 6 months | 1.553 (1.104 to 2.002) | 0.258 (-0.184 to 0.7) | - |
| Presenteeism at work* | 0.265 (0.179 to 0.351) | 0.385 (0.328 to 0.442) | 0.165 (0.067 to 0.262) |
| Constant | -3.493 (-5.117 to -1.869) | 0.309 (-0.982 to 1.601) | -3.035 (-5.148 to -0.923) |
| Random effect, sd(constant) | 0.789 (0.387 to 1.609) | 0.0001 (0 to 1.2E+157) | 0.6071 (0.2072 to 1.7785) |

* Coefficients refer to the effect for a one-unit increase in the variable e.g., per one-year increase in age

## Table S2: Prognostic model details (odds ratios, constant terms with variance, and shrinkage estimates) for models to predict any absence by 6 months, after adjustment for overfitting, for each IECV cycle.

| Variable | Excluding  BEEP | Excluding  KAPS | Excluding  STEMS | Excluding  STarT MSK-MT | Excluding  STarT MSK-pilot |
| --- | --- | --- | --- | --- | --- |
| Age* | 0.984 | 0.984 | 0.986 | 0.975 | 0.992 |
| Female | 0.777 | 0.694 | 0.749 | 0.801 | 0.659 |
| Multisite pain | 0.815 | 0.701 | 1.11 | 0.862 | 0.893 |
| Baseline pain score* | 1.127 | 1.182 | 1.084 | 1.148 | 1.14 |
| Pain duration |  |  |  |  |  |
| <3 months | ref | ref | ref | ref | ref |
| 3-6 months | 1.354 | 1.501 | 1.69 | 0.694 | 1.471 |
| 7-12 months | 1.004 | 0.889 | 0.874 | 1.189 | 1.146 |
| >12 months | 1.528 | 1.486 | 1.658 | 1.716 | 1.425 |
| Job type |  |  |  |  |  |
| Professional/managerial | ref | ref | ref | ref | ref |
| Intermediate | 0.975 | 0.998 | 0.788 | 1.133 | 1.061 |
| Manual | 0.784 | 0.572 | 0.809 | 0.866 | 0.694 |
| Anxiety/depression | 1.395 | 1.359 | 1.575 | 1.521 | 1.374 |
| Other comorbidity | 0.985 | 0.89 | 1.23 | 1.287 | 0.904 |
| Work absence in previous 6 months | 4.427 | 5.826 | 4.457 | 2.255 | 4.07 |
| Presenteeism at work* | 1.288 | 1.292 | 1.261 | 1.245 | 1.305 |
| Constant** | -3.452 | -2.851 | -3.357 | -3.246 | -3.741 |
| Random effect**, sd(constant) | 0.822 | 0.598 | 0.852 | 0.665 | 0.714 |
| *Shrinkage* | 0.942 | 0.945 | 0.932 | 0.875 | 0.930 |

* Odds ratios refer to the effect for a one-unit increase in the variable e.g., per one-year increase in age

** Constant and random effects terms were re-estimated after adjustment for optimism to maintain overall model calibration.

## Figure S1a: Apparent calibration of 6-month absence model (after adjustment for overfitting), by study subgroups, and on average across all data

| BEEP 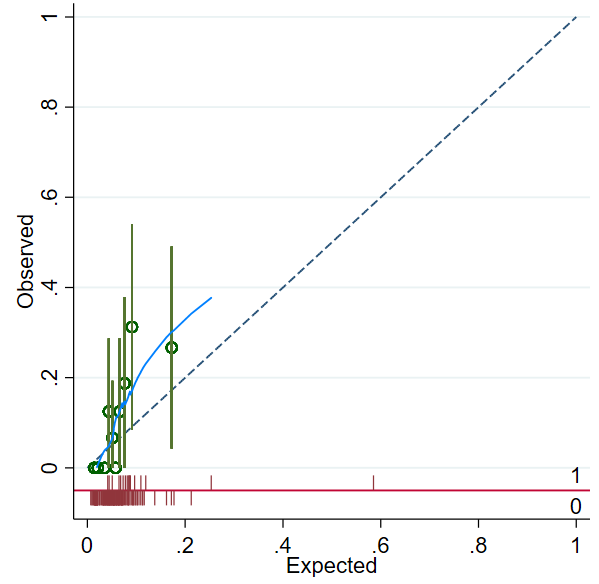 | KAPS 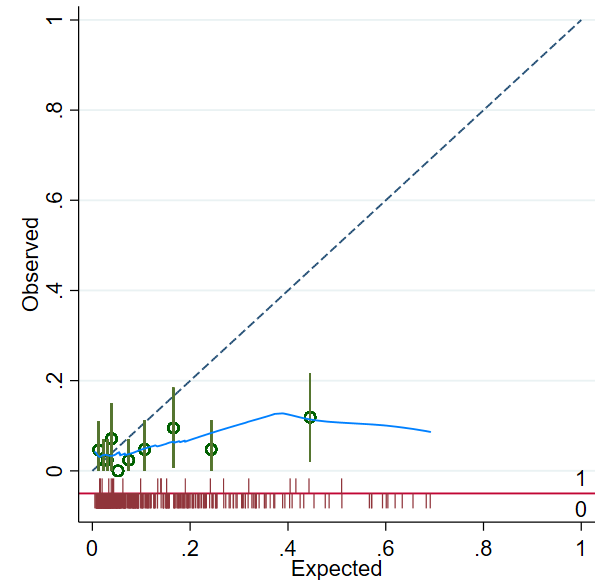 |
| --- | --- |
| STEMS 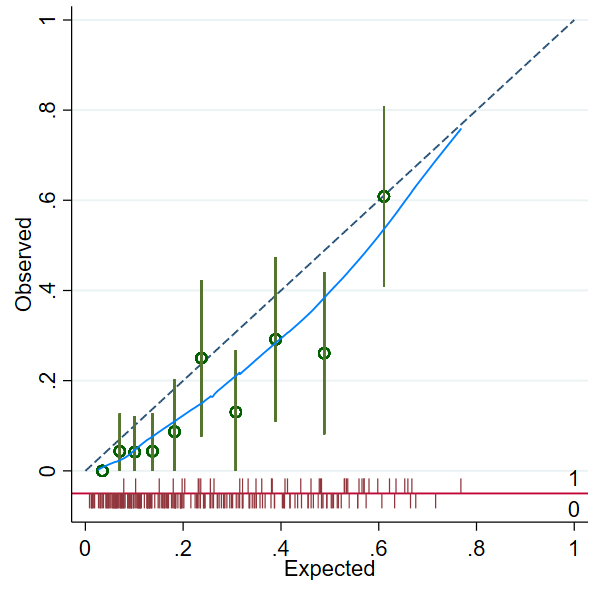 | STarT MSK-MT 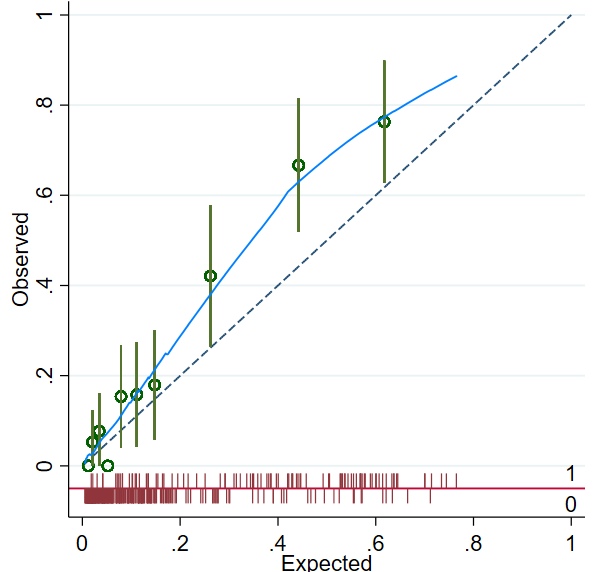 |
| STarT MSK-pilot 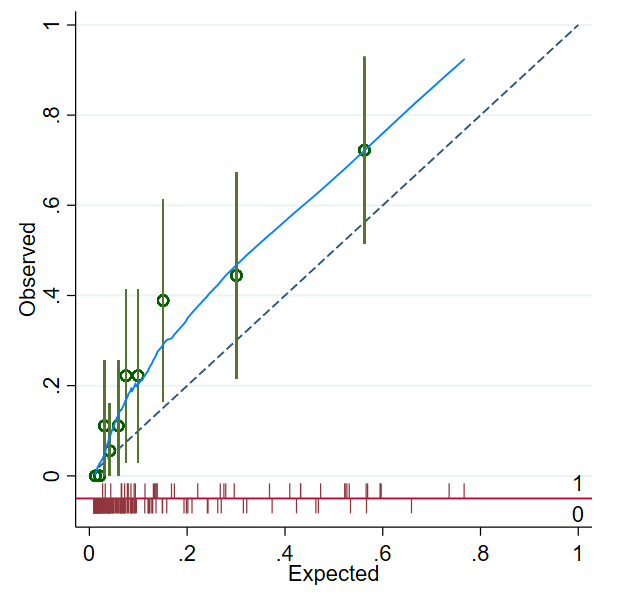 | All data 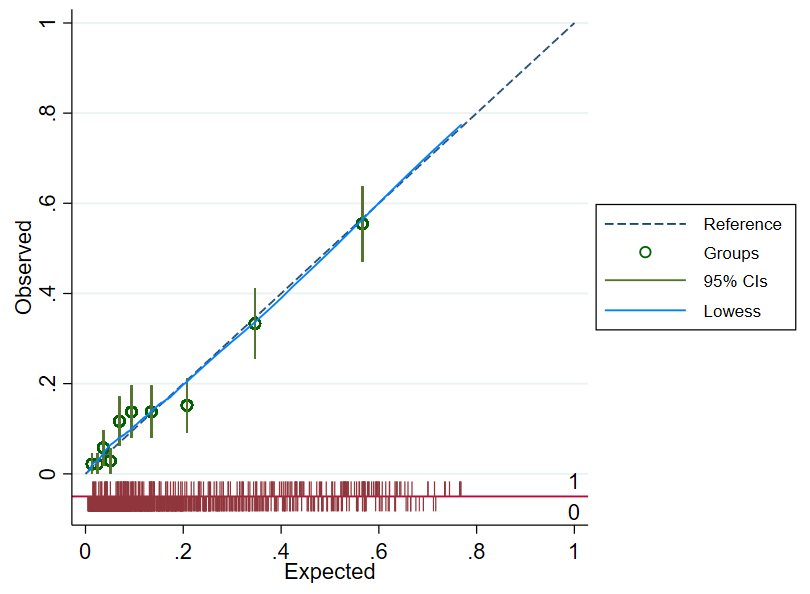 |

## Figure S1b: Apparent calibration of 6-month presenteeism model (after adjustment for overfitting), by study subgroups, and on average across all data

| STEMS 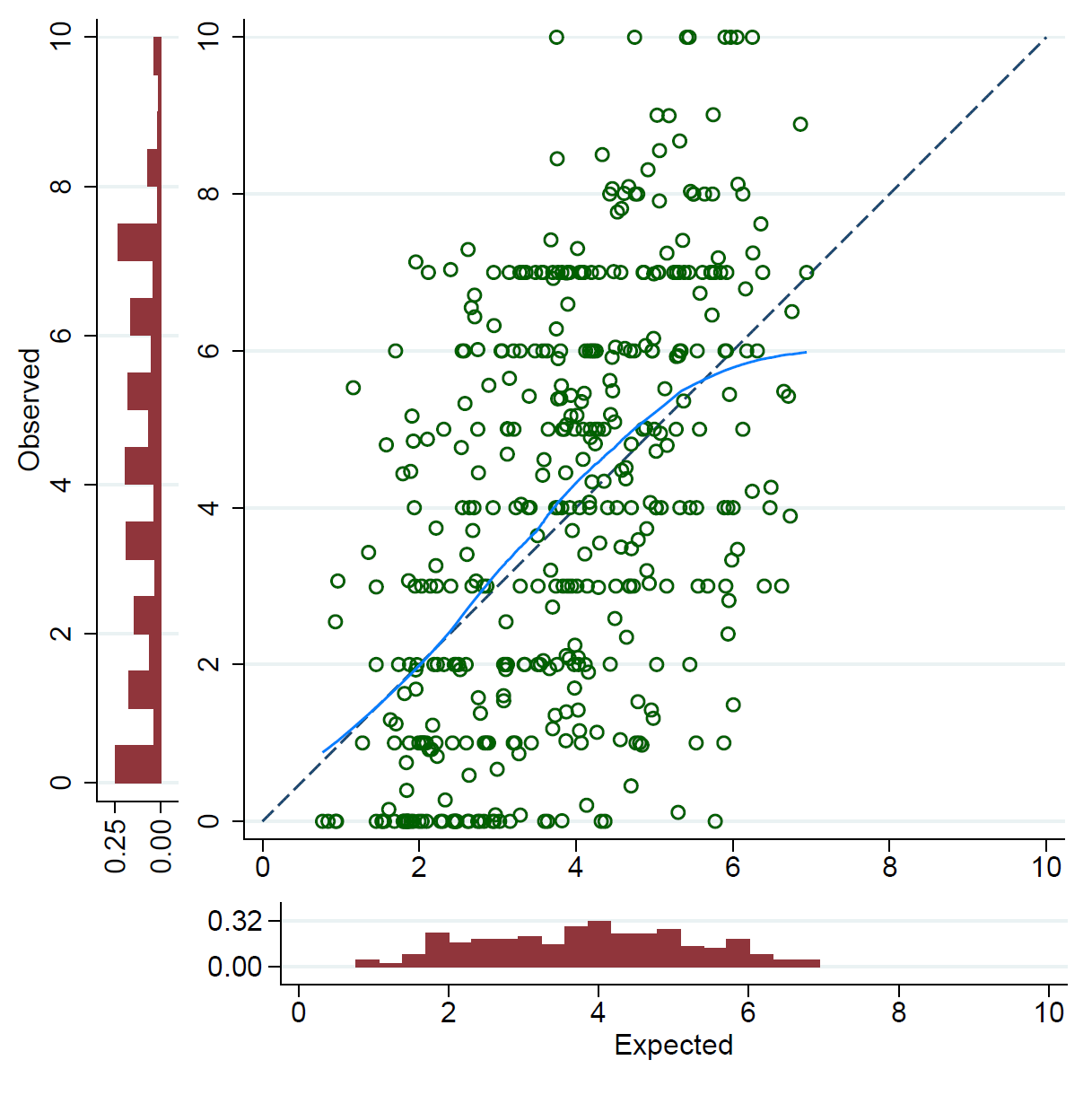 | STarT MSK-MT 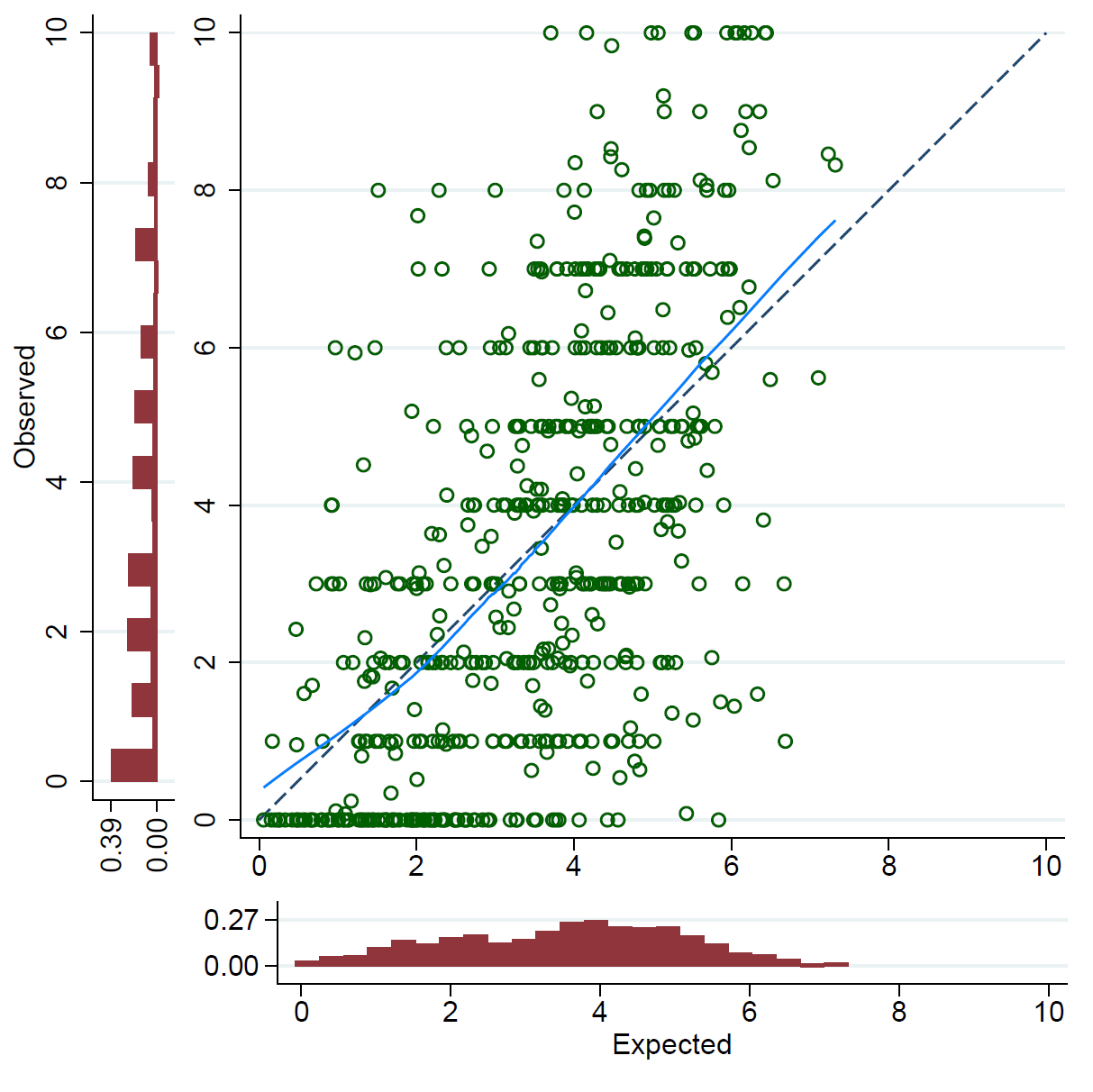 |
| --- | --- |
| STarT MSK-pilot 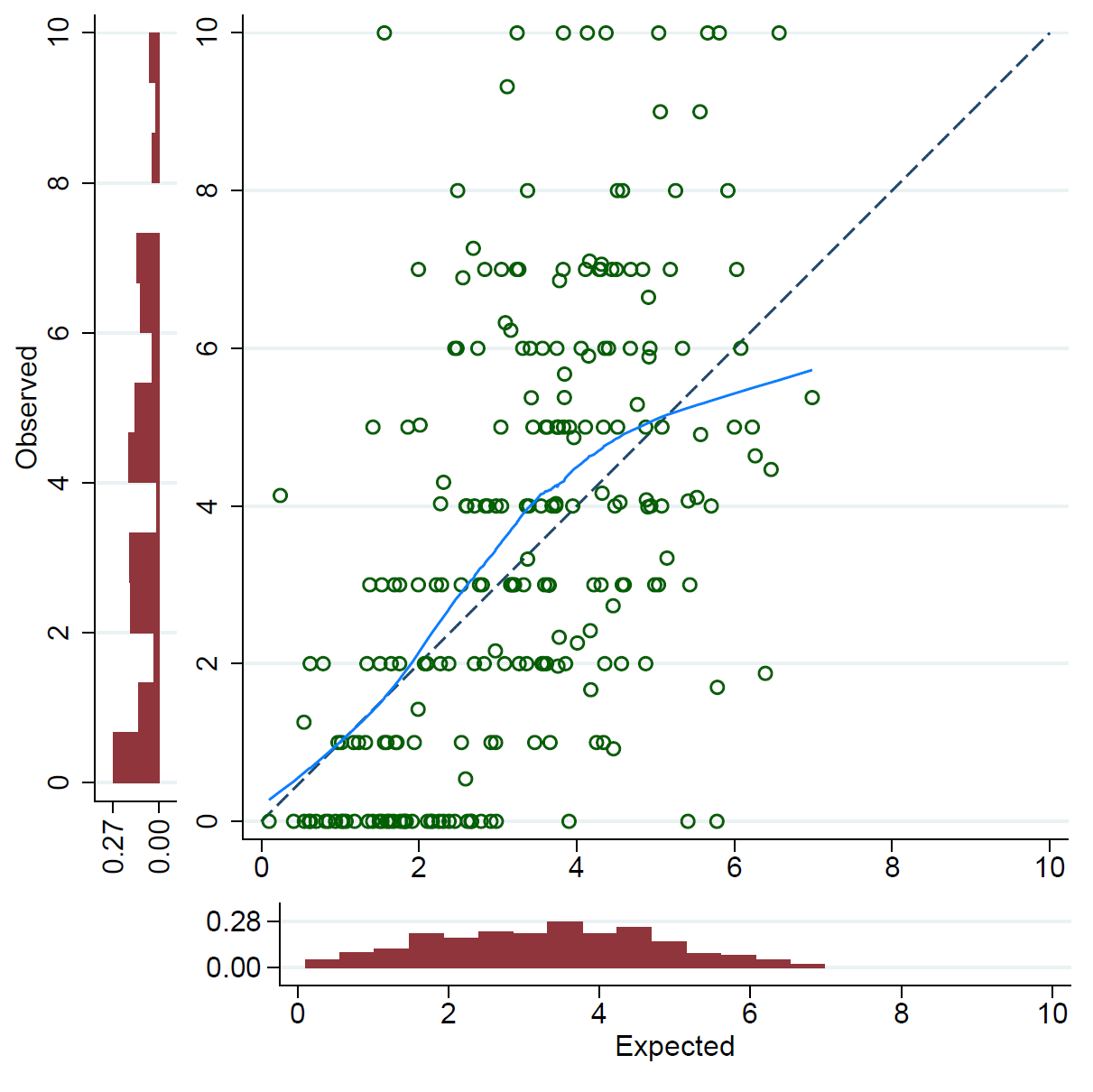 | All data 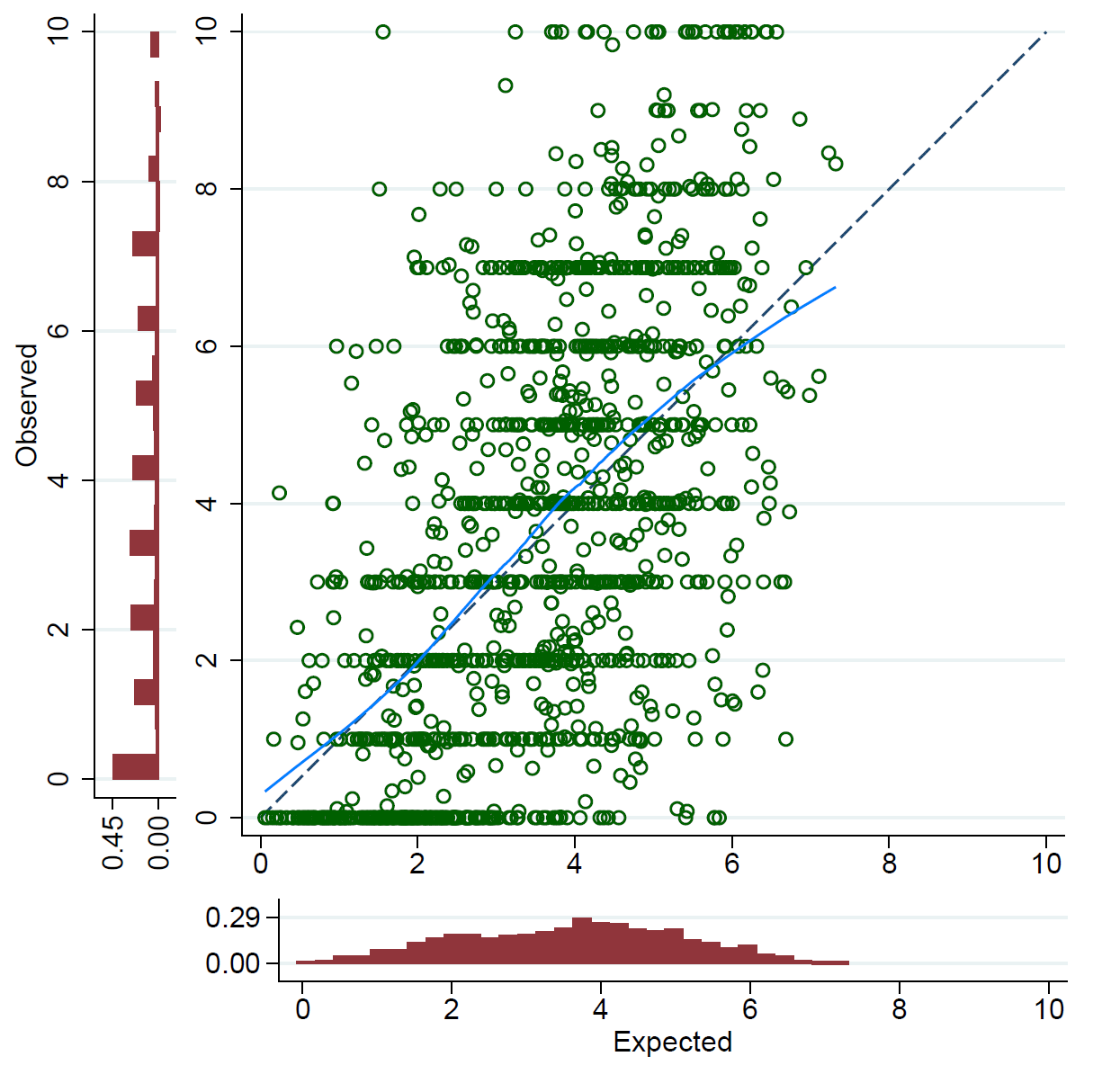 |

## Figure S1c: Apparent calibration of 12-month absence model (after adjustment for overfitting), by study subgroups, and on average across all data

| STEMS  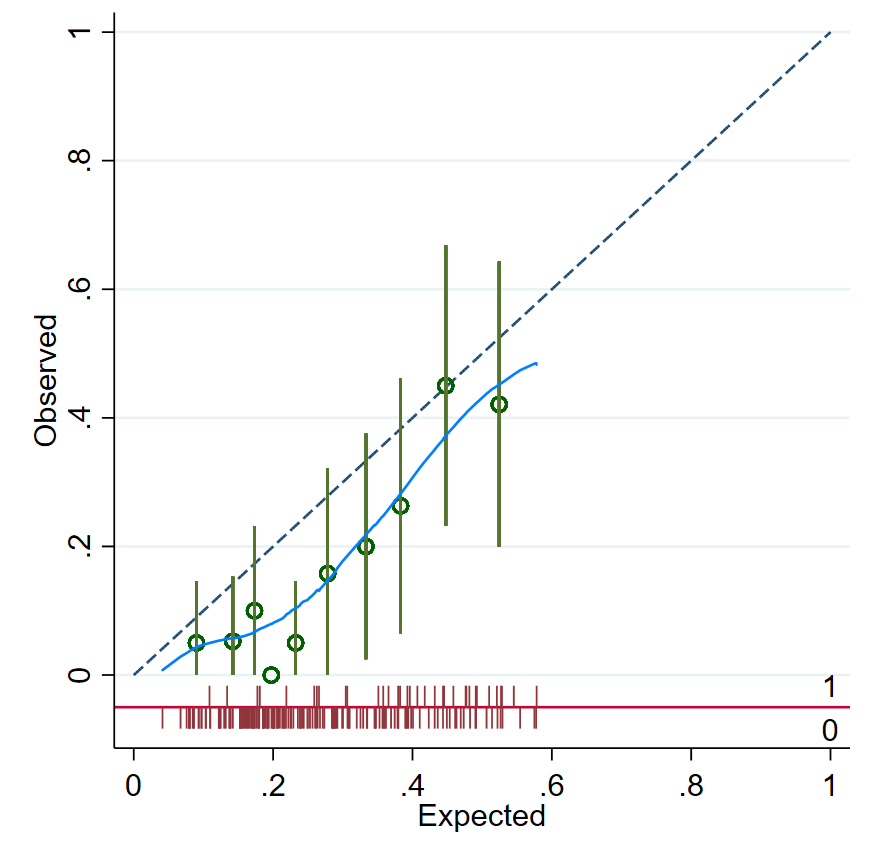 | SWAP  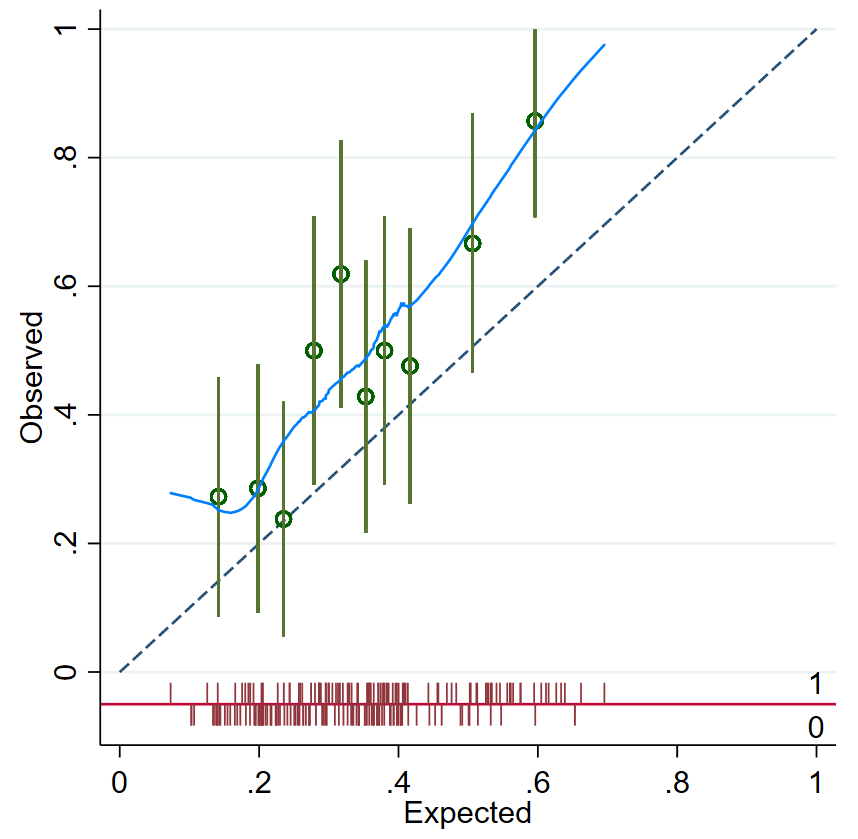 |
| --- | --- |
| All data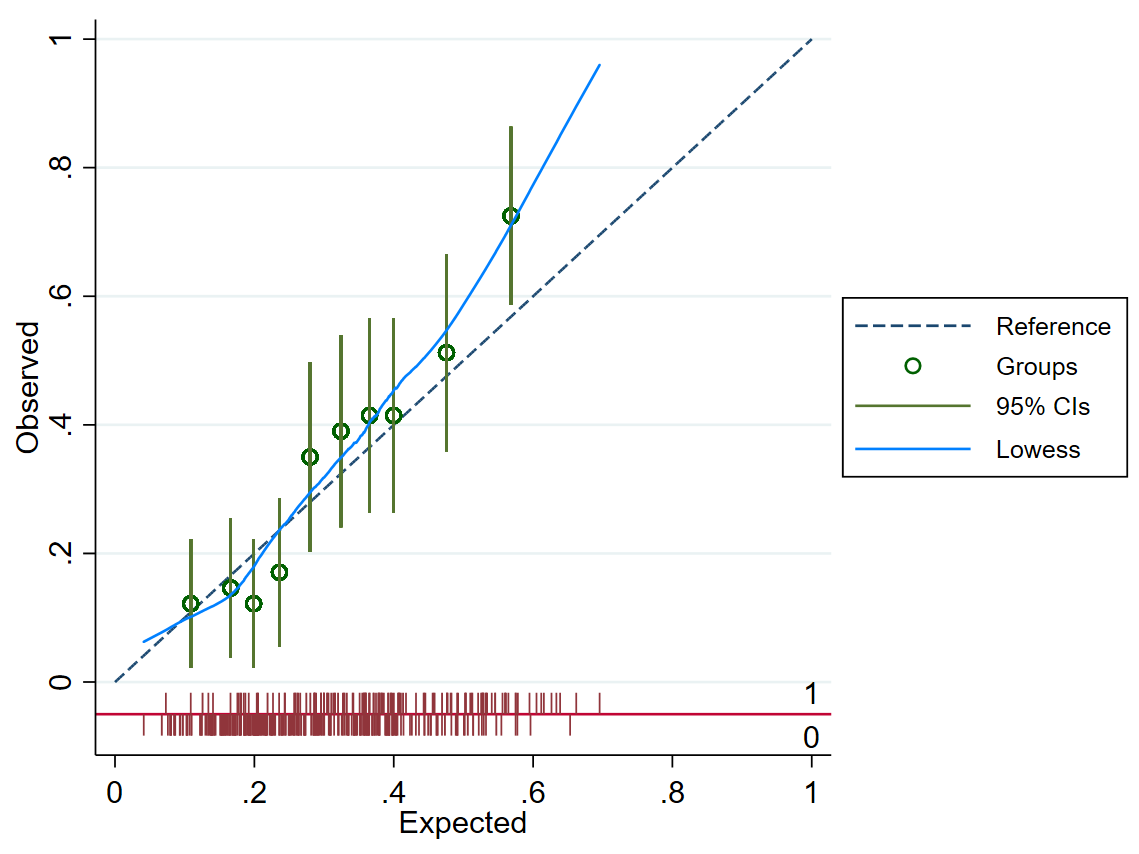 | |

## Figure S2a: Distribution of predicted probabilities for absence at 6 months (by outcome status) for each study in the IECV cycle in which it was excluded from model development, and of the final shrunken model in all datasets combined

| BEEP  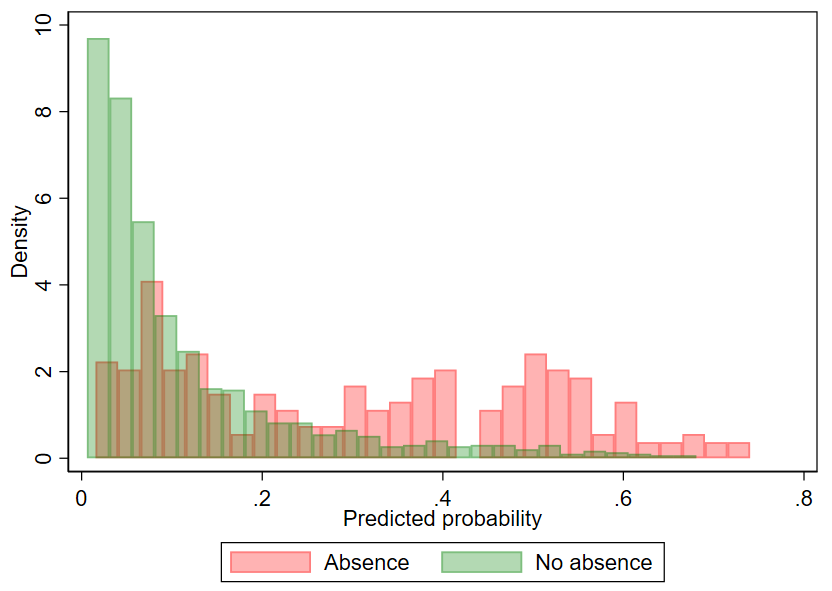 | KAPS  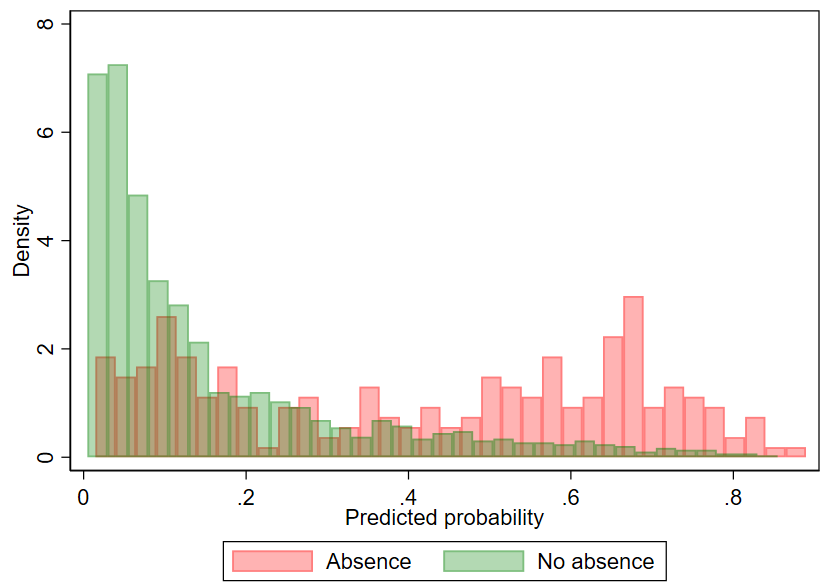 |
| --- | --- |
| STEMS  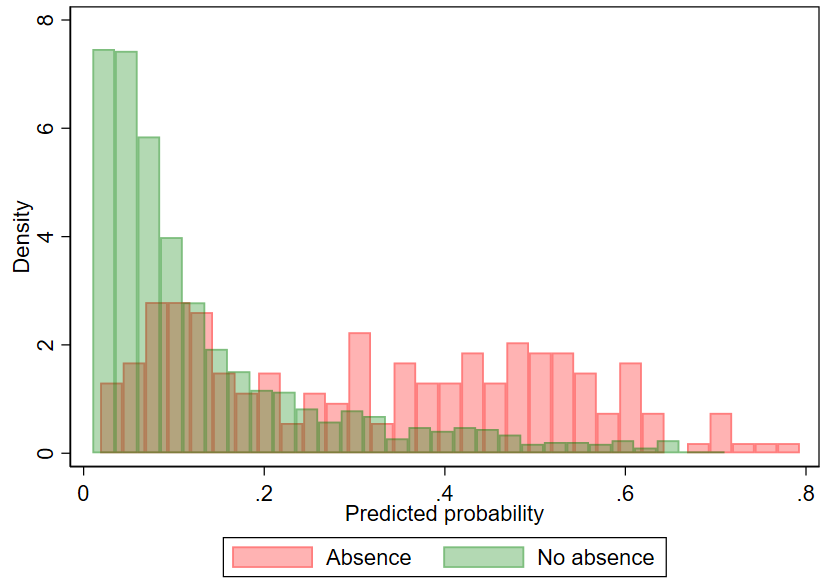 | STarT MSK-MT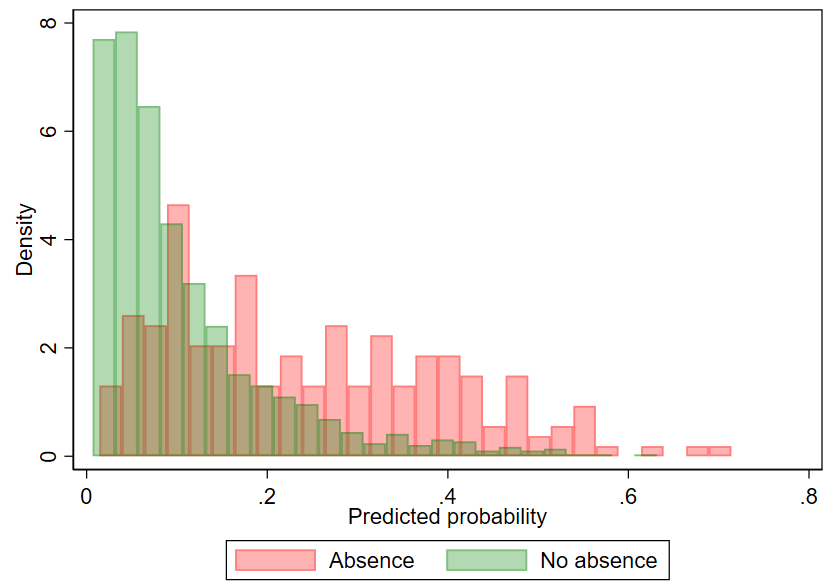 |
| STarT MSK-pilot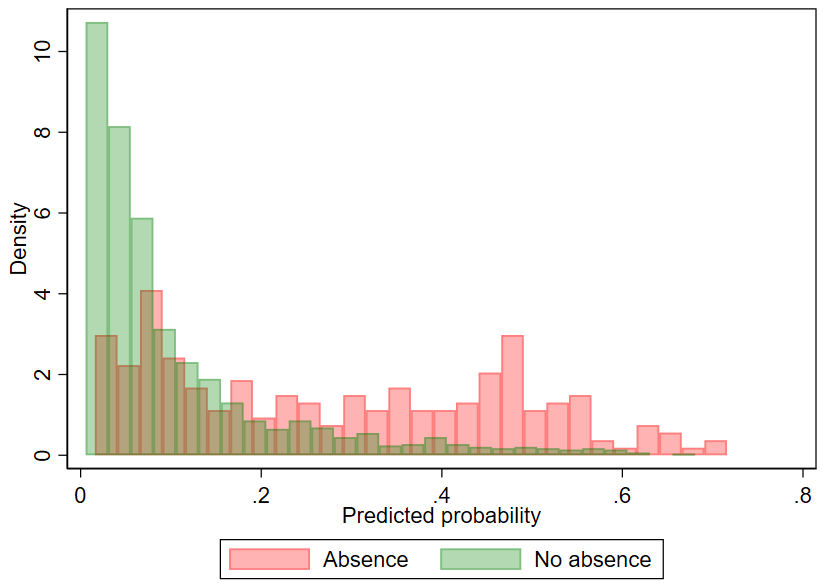 | All data 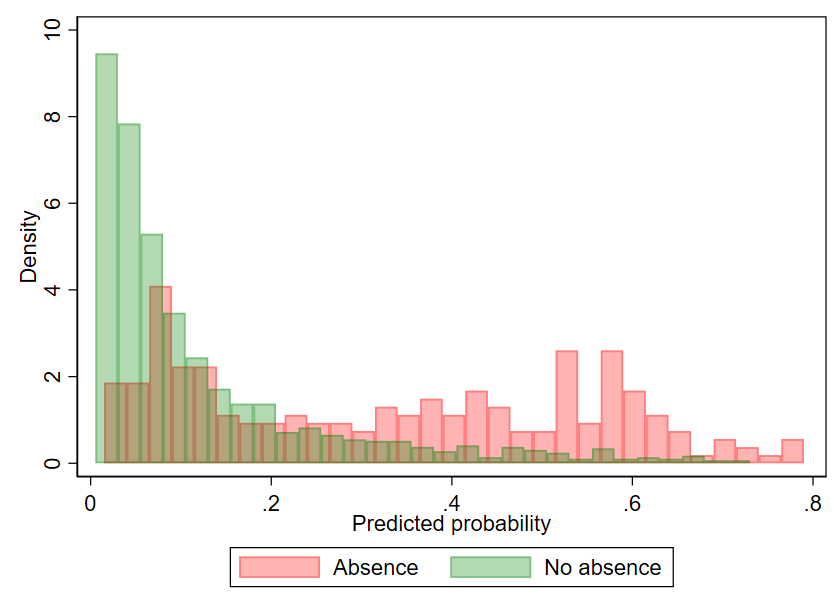 |

## Figure S2b: Distribution of predicted values for presenteeism at 6 months for each study in the IECV cycle in which it was excluded from model development, and of the final shrunken model in all datasets combined

| STEMS 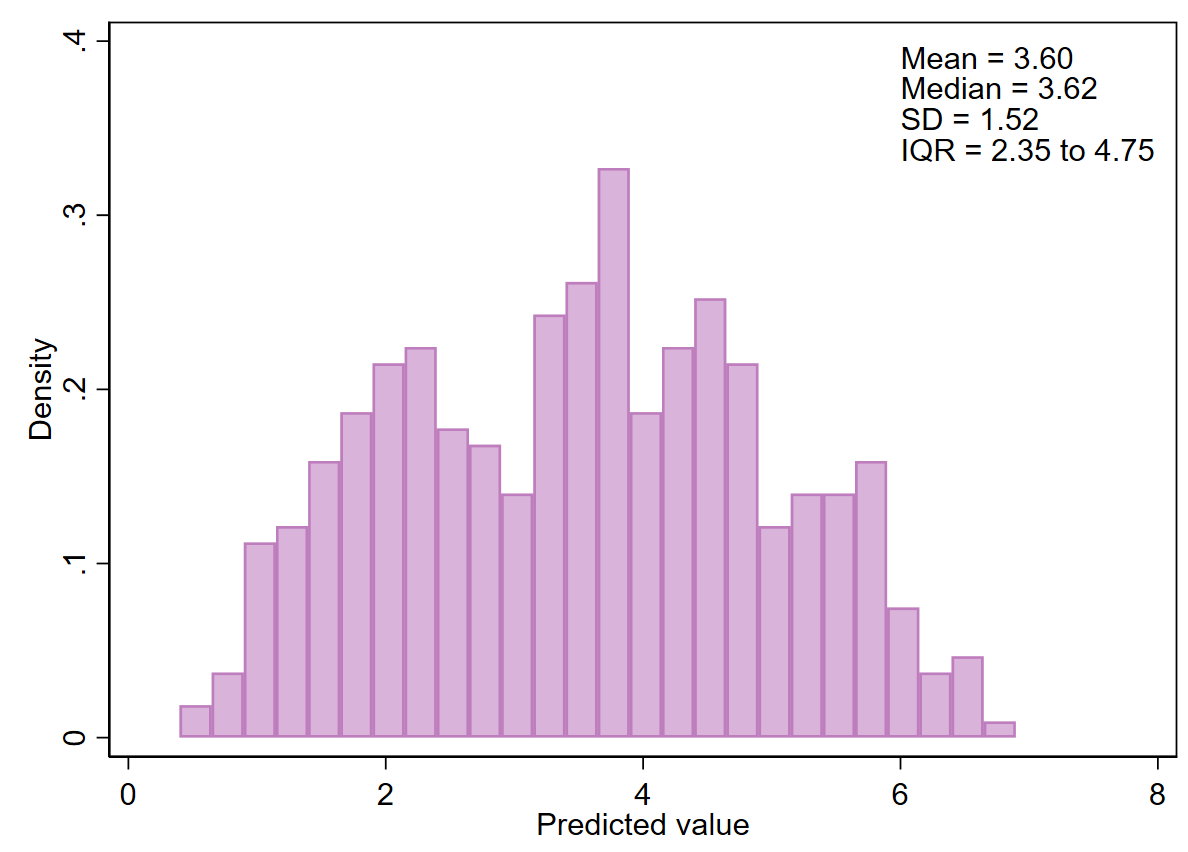 | STarT MSK-MT 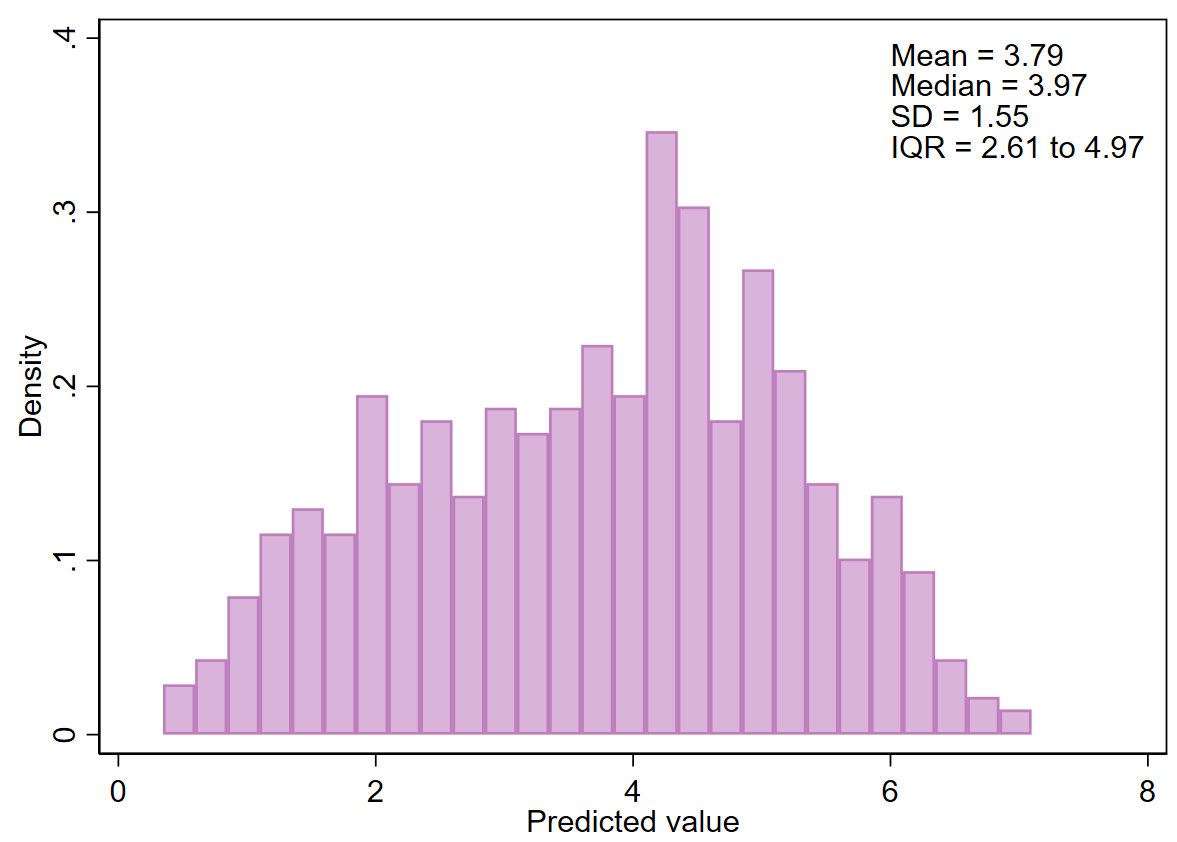 |
| --- | --- |
| STarT MSK-pilot 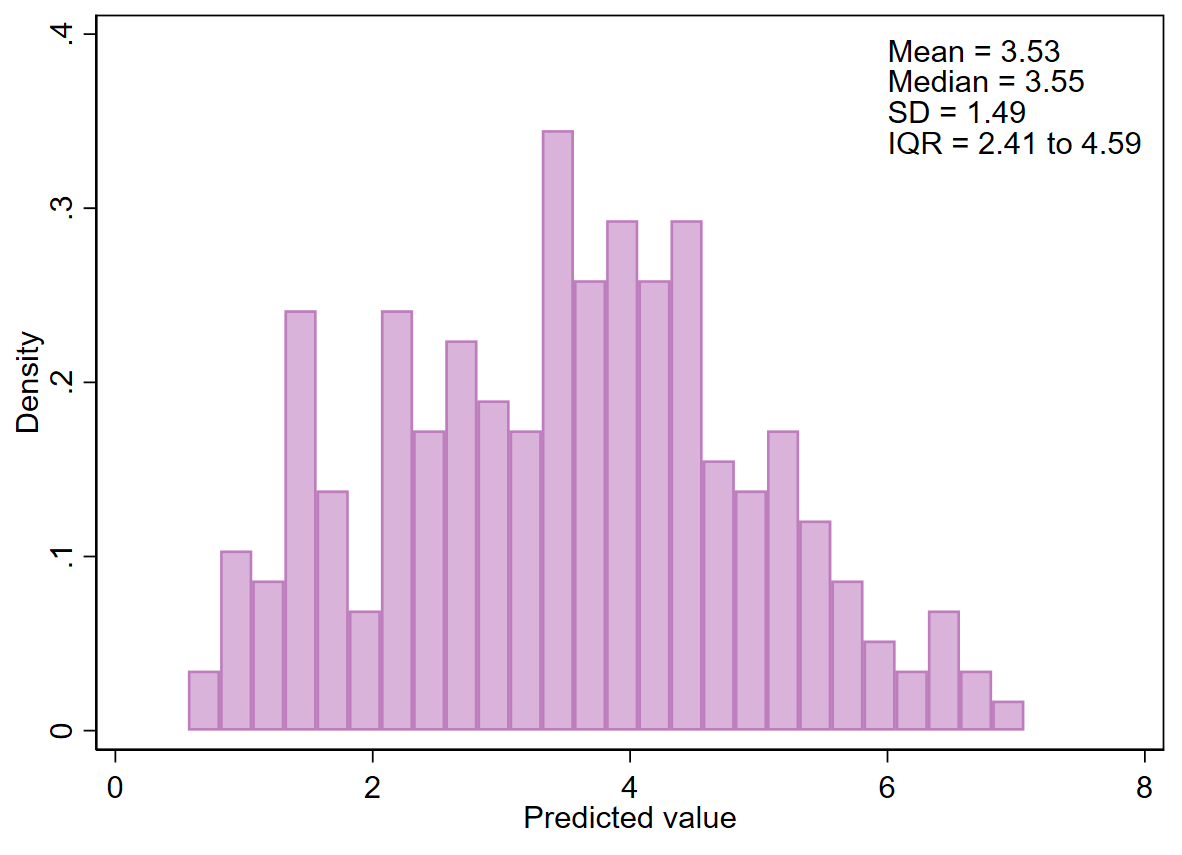 | All data 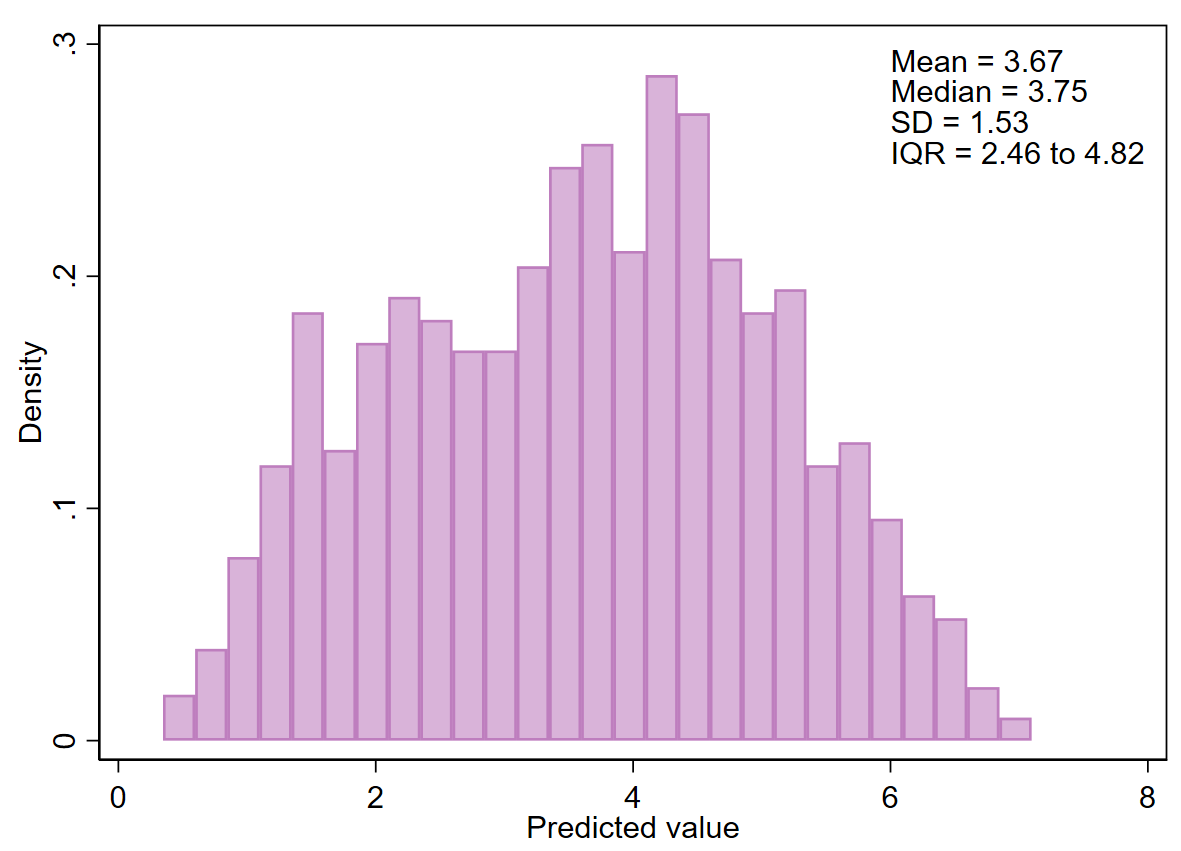 |

## Figure S2c: Distribution of predicted values for probability of absence at 12 months of the final shrunken model, for each study subgroup and in both datasets combined

| STEMS  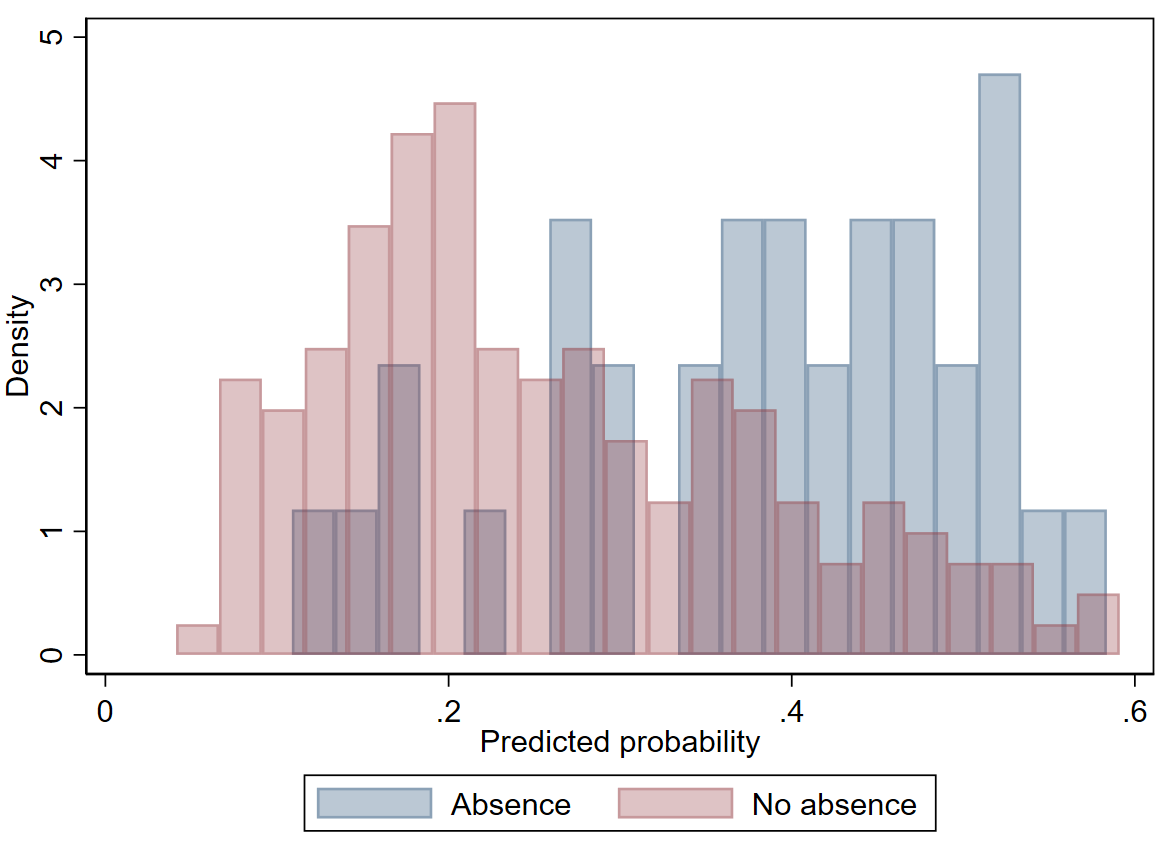 | SWAP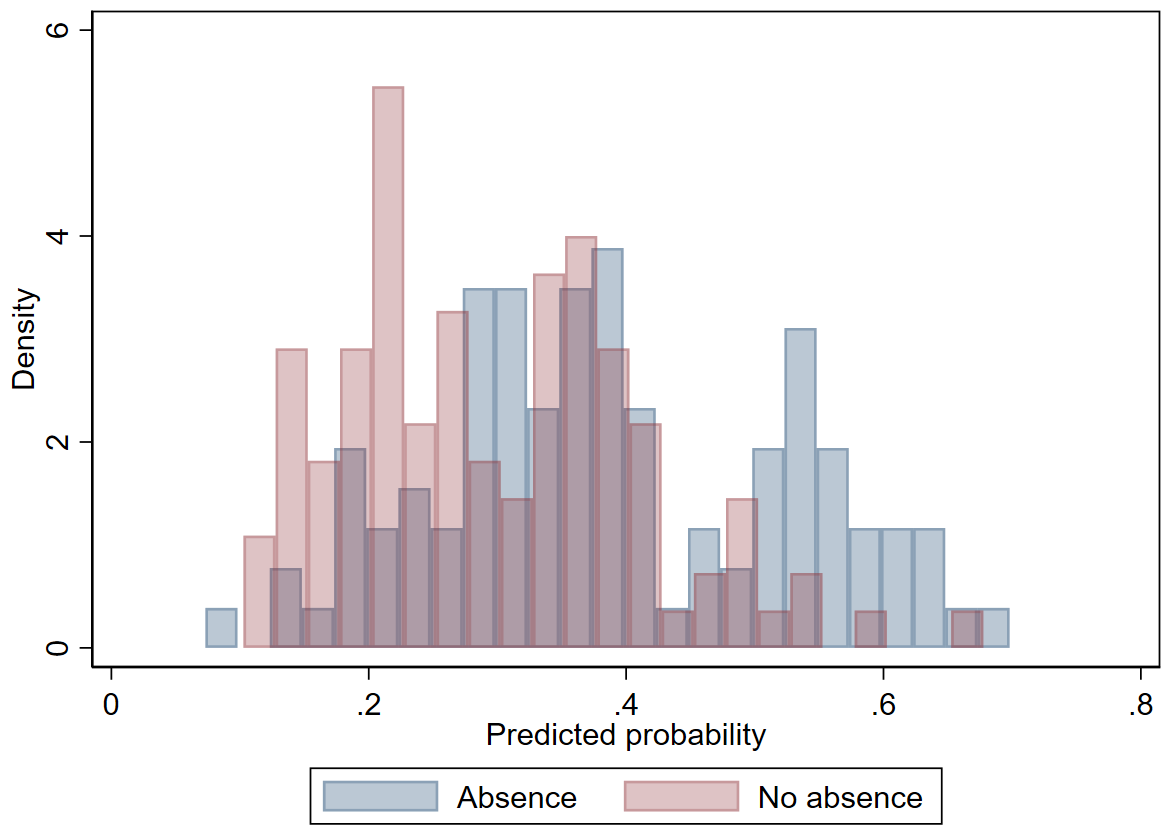 |
| --- | --- |
| All data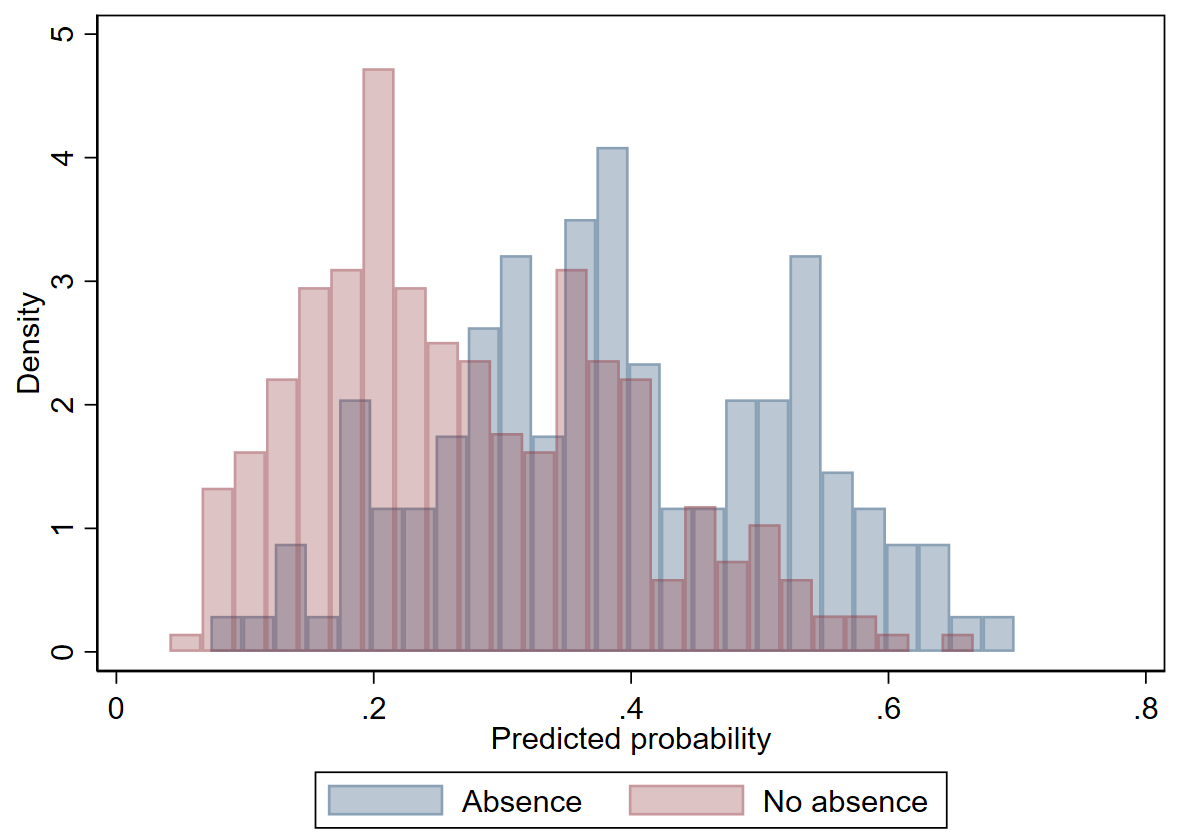 |  |

## Figure S3a: Decision curves for models to predict 6 and 12-month absence. Each plot shows the performance of the final shrunken model, when applied across all studies combined (without accounting for clustering of data by study). Clinically relevant risk threshold range, defined *a priori* based on clinical expert opinion and previous literature as being decision thresholds anywhere between 0.26 and 0.49, is highlighted.

| 6m absence | 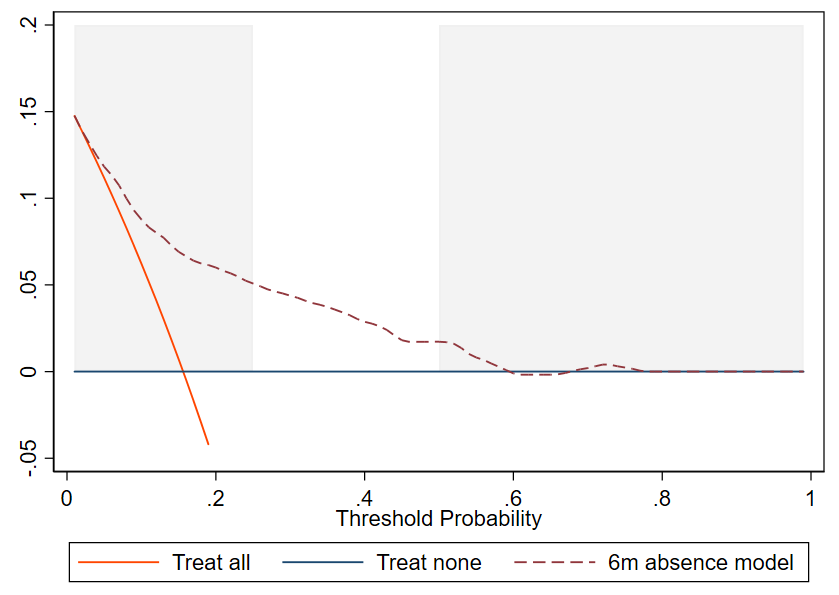 |
| --- | --- |
| 12m absence | 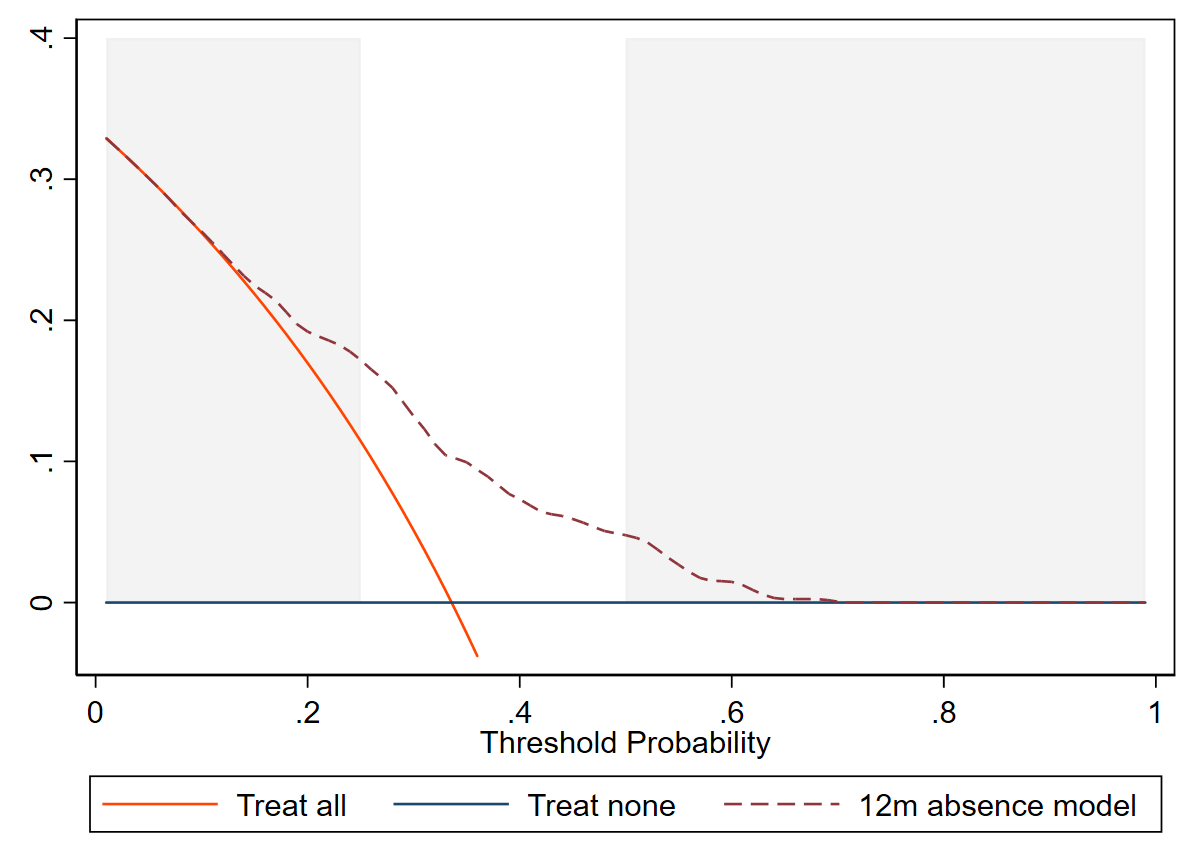 |

Net benefit analysis indicated potential clinical utility of using the 6-month absence model with decision thresholds within the pre-specified range (26% to 49%, as defined based on clinical expert opinion and previous literature), which was evident across all IECV cycles as well as in the full, combined data from all studies (see figure 1 and supplementary figure S3a). For example, when assessing net benefit across all studies, using a treatment decision threshold of 26% (those with predicted risks of 25% or lower assigned to usual care, whilst those with a risk of 26% or higher receive additional support) gave an estimated net benefit of 0.049. This corresponds to 49 true absences identified, over-and-above those offset by incorrectly identified absences, per 1000 people using the model. At this same threshold, the “treat none” (usual care for everyone) approach gave a net benefit of zero (no benefit or harm overall), whilst the “treat all” approach resulted in net harm across the population (the harm from the number of incorrectly identified absences outweighing the benefit from the correctly identified absences). At a treatment decision threshold of 49% (only those with absence risks of at least 50% receiving any intervention), the net benefit of 0.015 suggests that only 15 people would be correctly offered additional support, after offsetting incorrectly identified absences, for each 1000 people in whom the model was applied.

## Figure S3b: Decision curves for model to predict the probability of absence at 6 months for each study in the IECV cycle in which it was excluded from model development, and of the final shrunken model in all datasets combined. Clinically relevant risk threshold range between 0.25 and 0.5

| BEEP  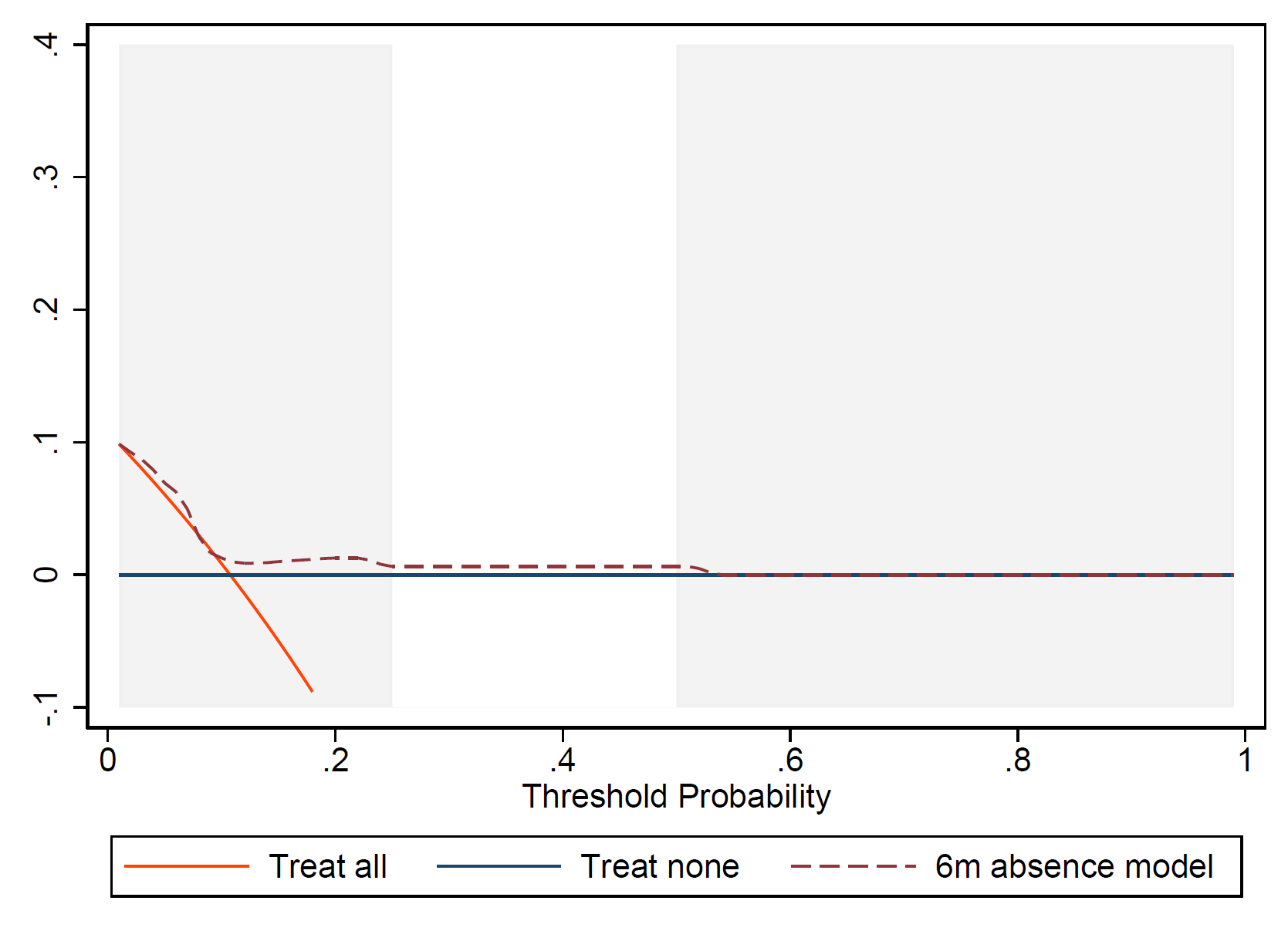 | KAPS  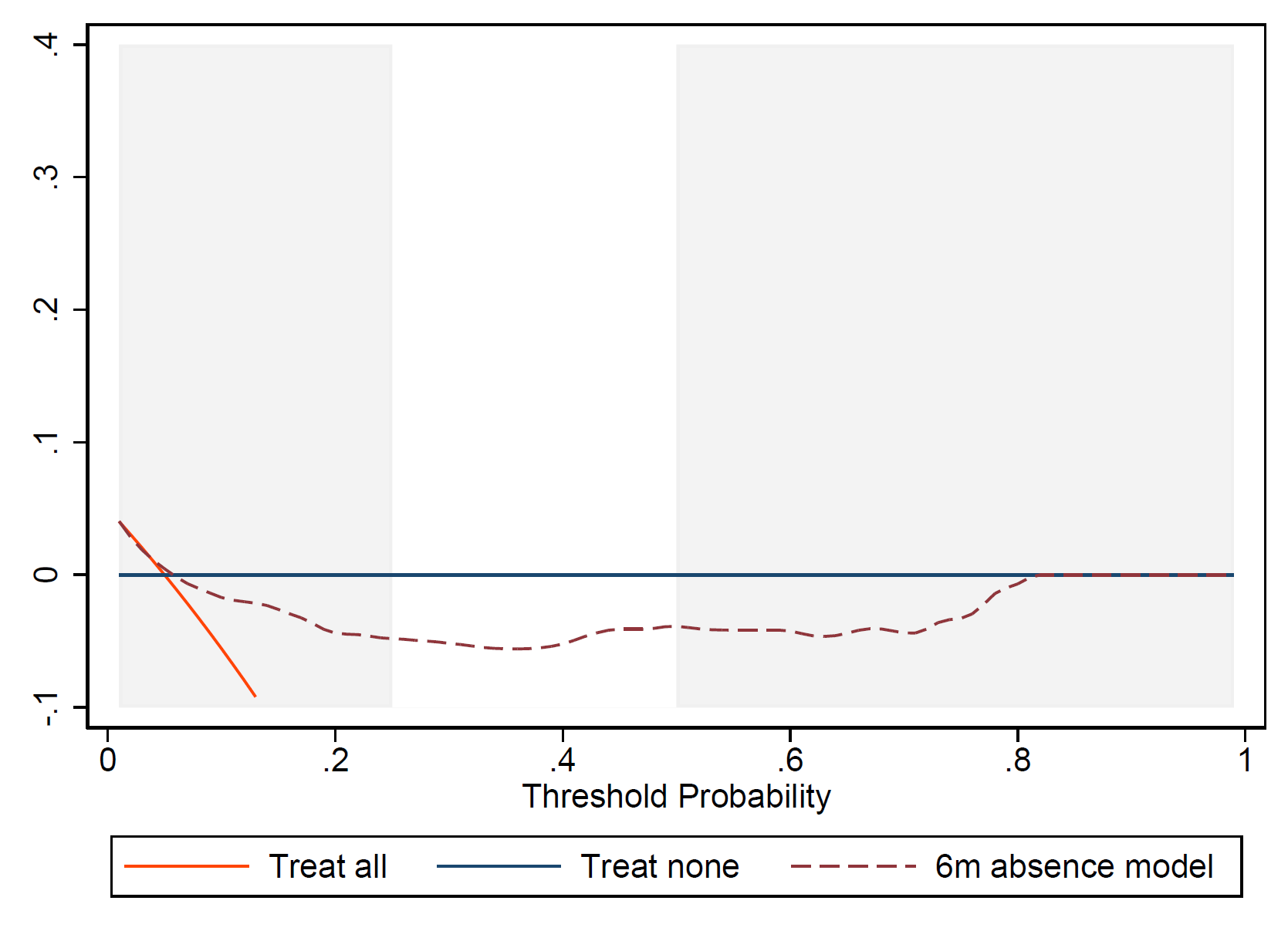 |
| --- | --- |
| STEMS  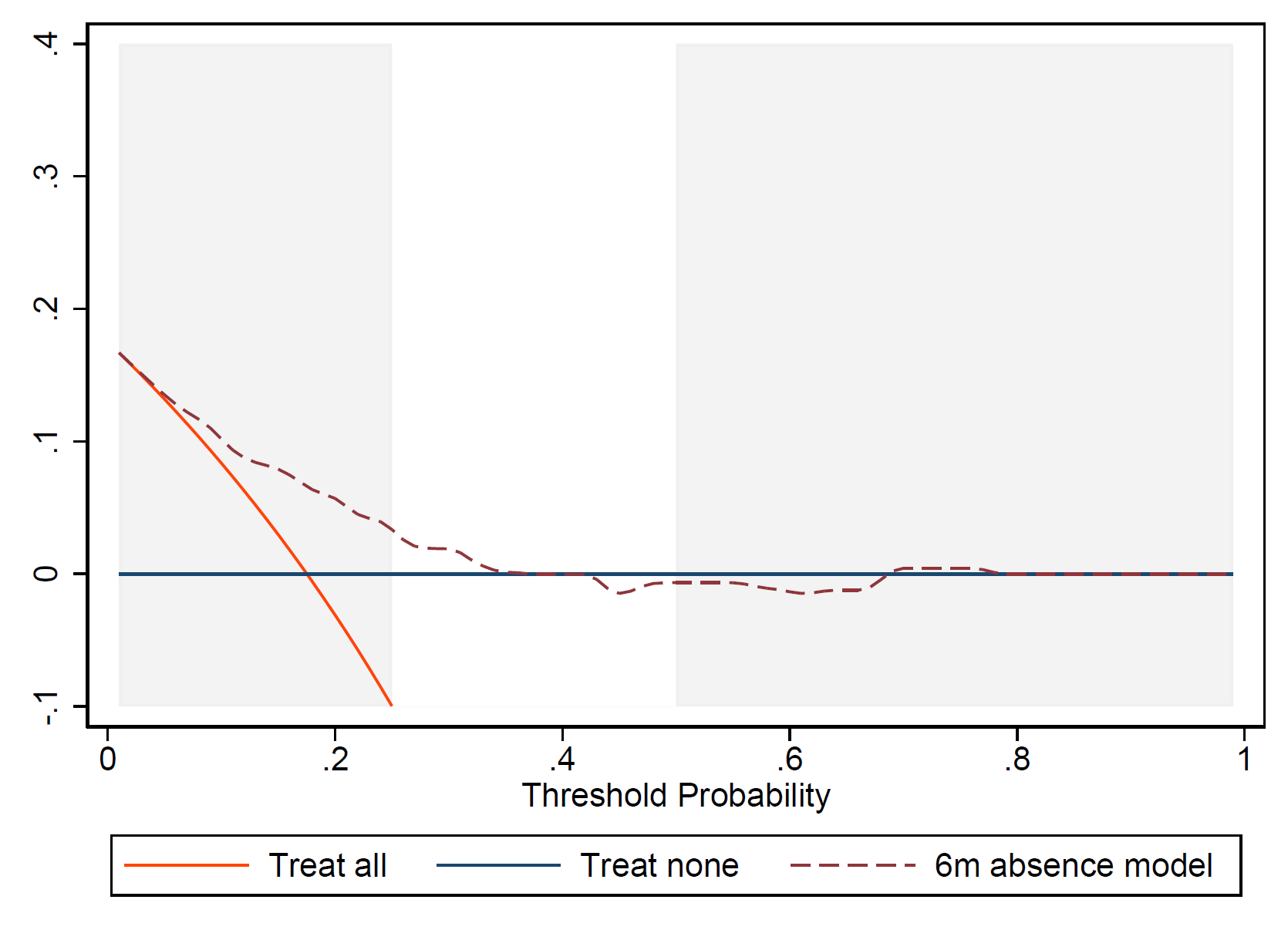 | STarT MSK-MT  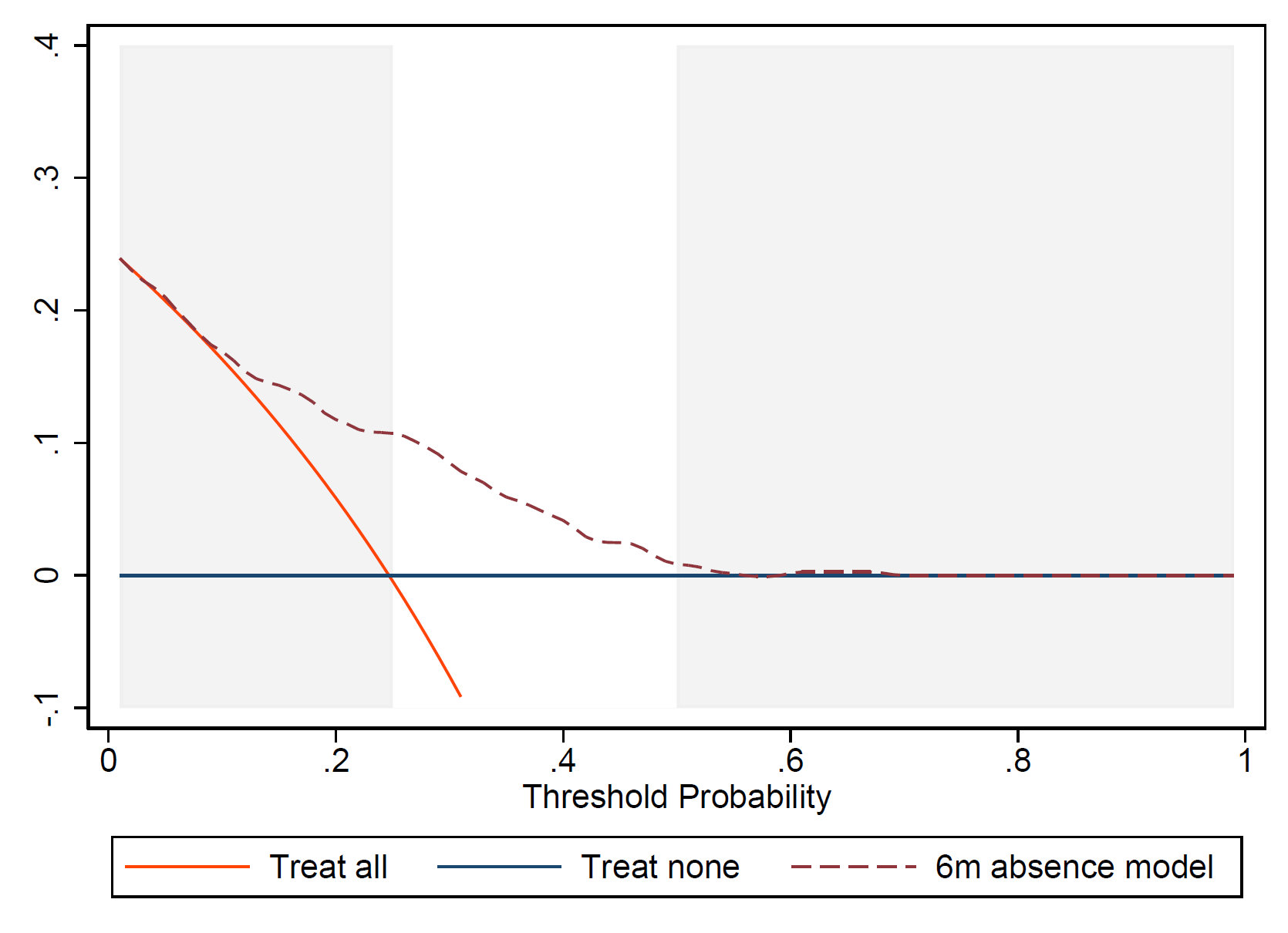 |
| STarT MSK-pilot  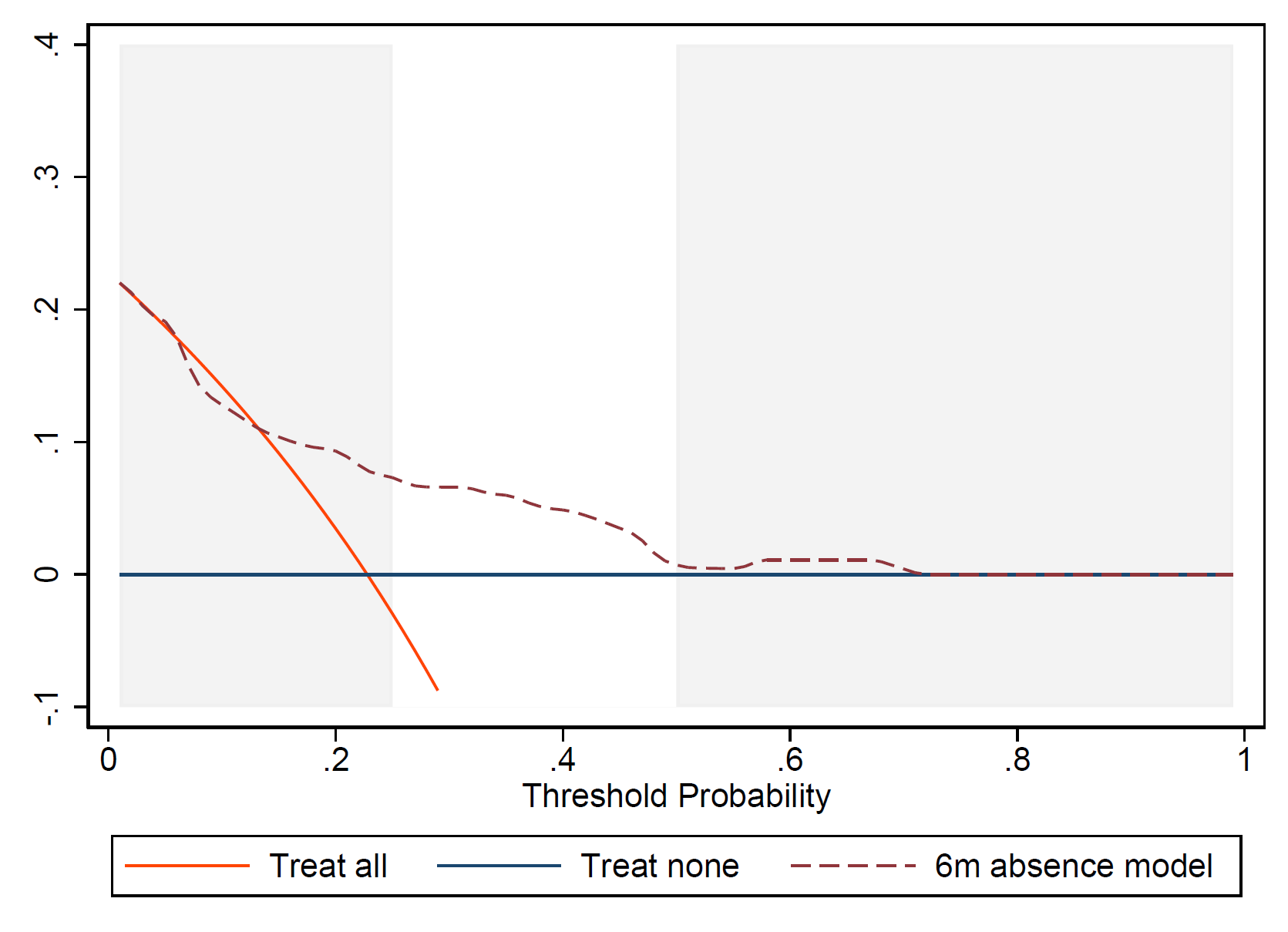 | All data  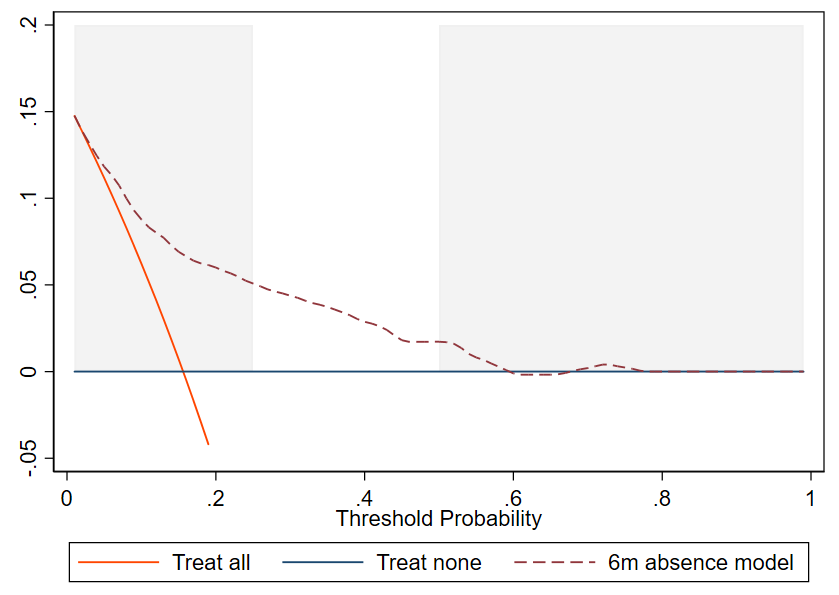 |

## Figure S3c: Decision curves for model to predict the probability of absence at 12 months of the final shrunken model, for each study subgroup and in both datasets combined. Clinically relevant risk threshold range between 0.25 and 0.5.

| STEMS  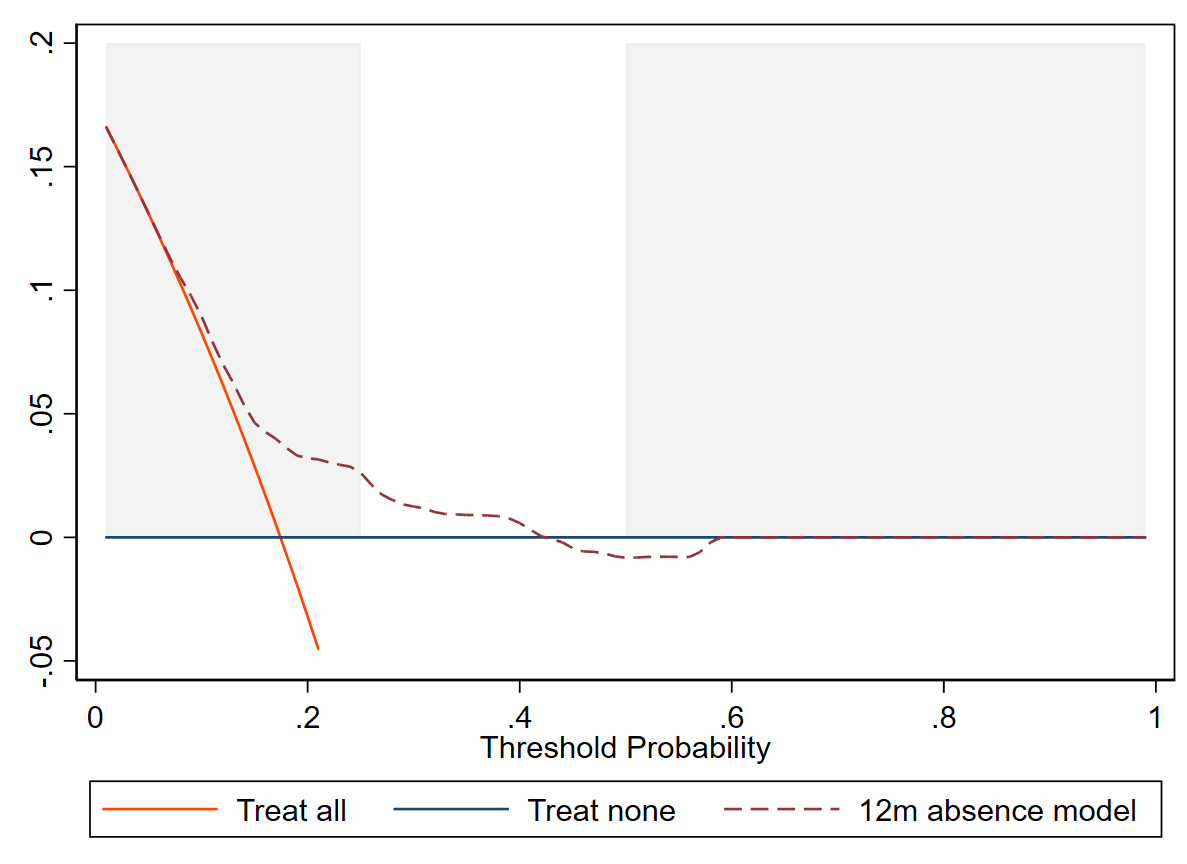 | SWAP  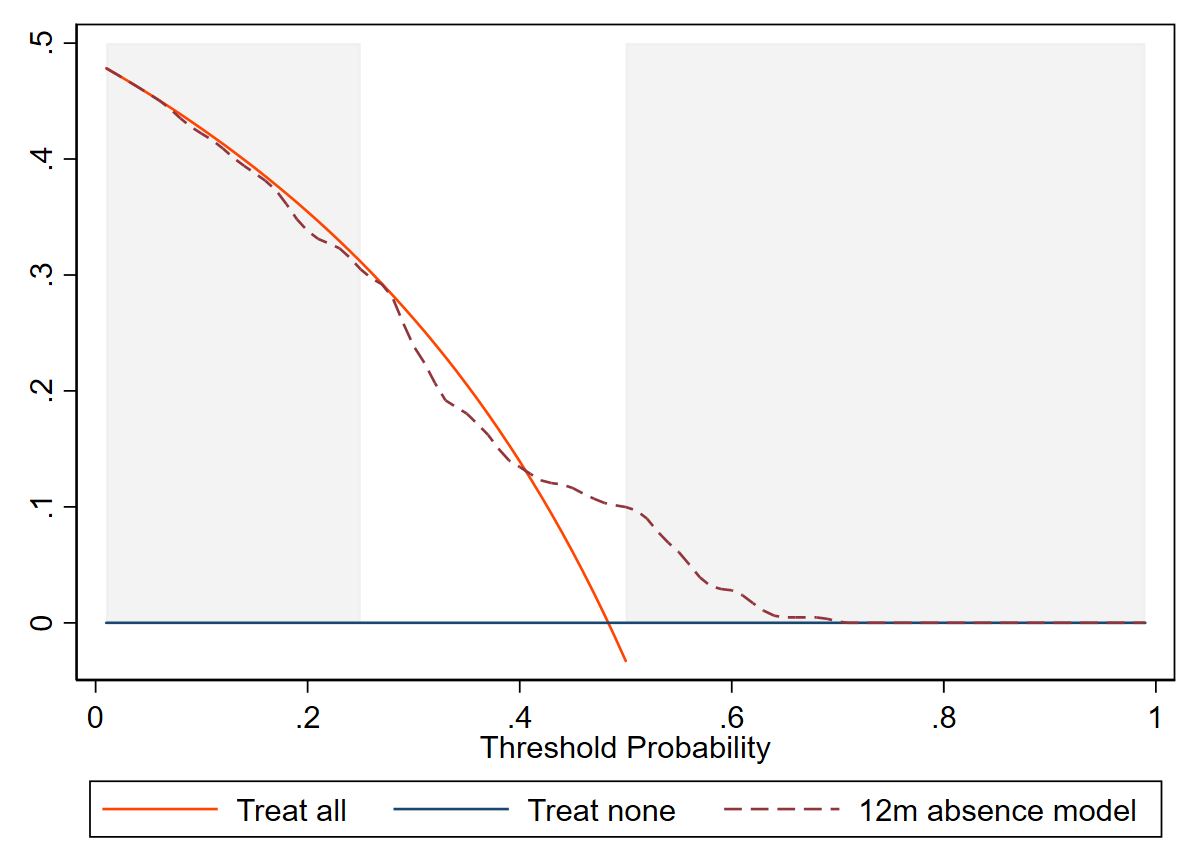 |
| --- | --- |
| All data  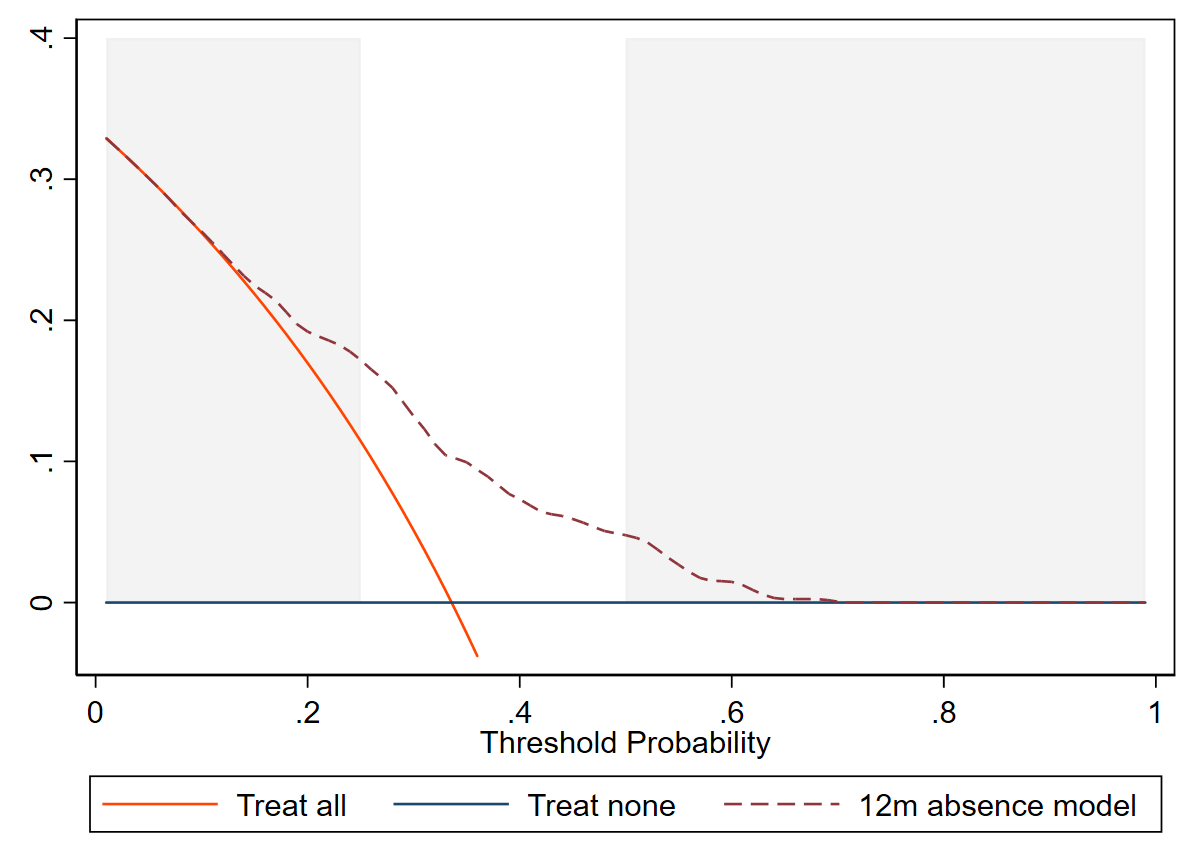 | |

## Figure S4a: Forest plots of pooled model performance estimates for the 6-month absence model, across IECV cycles


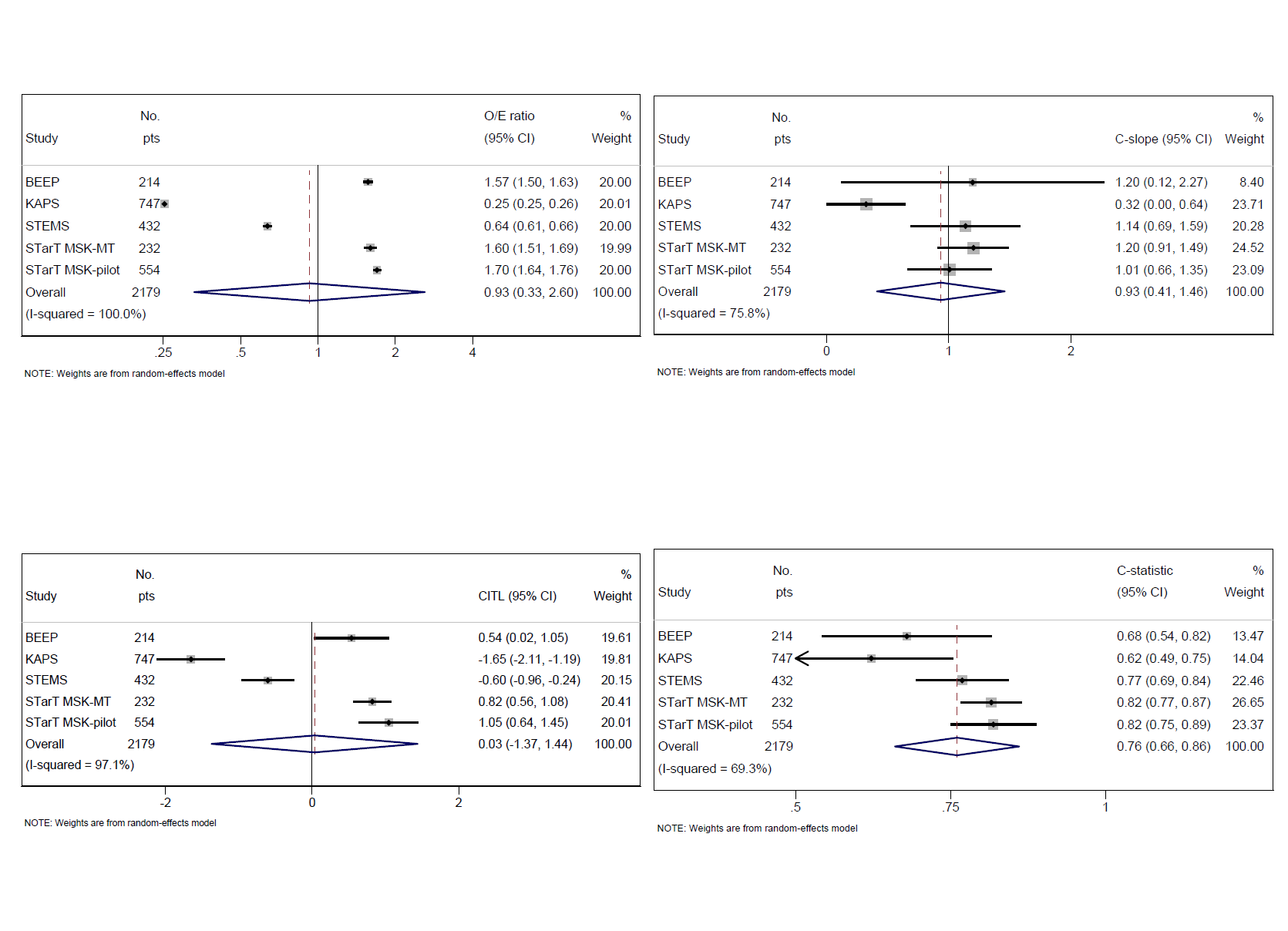


## Figure S4b: Forest plots of pooled model calibration estimates for the 6-month presenteeism model, across IECV cycles


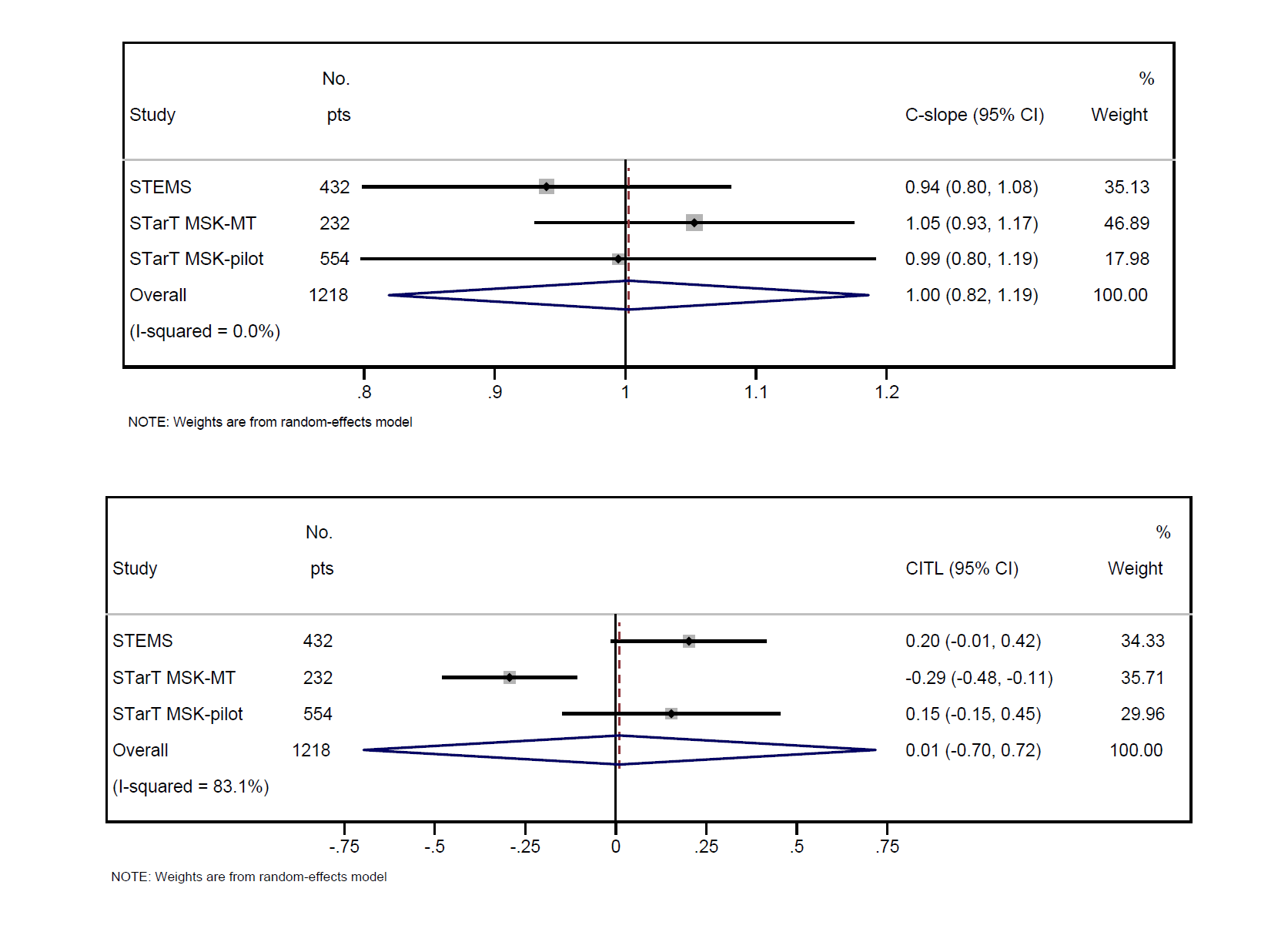


## Figure S5a: External calibration of 6-month absence model (after adjustment for overfitting), for each study in the IECV cycle in which it was excluded from model development

| BEEP  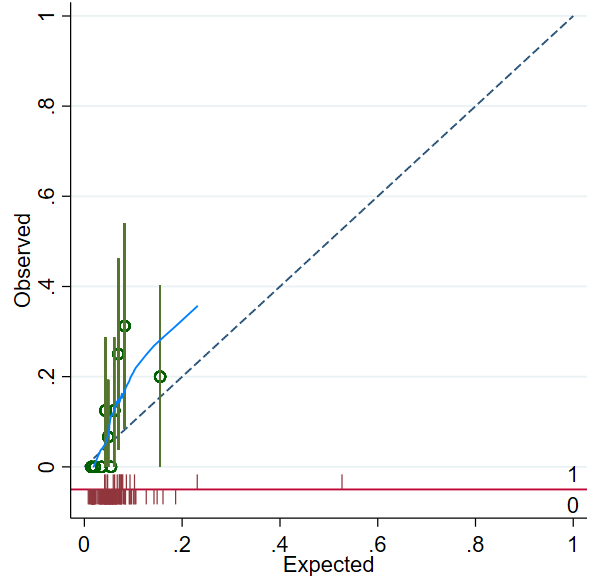 | KAPS  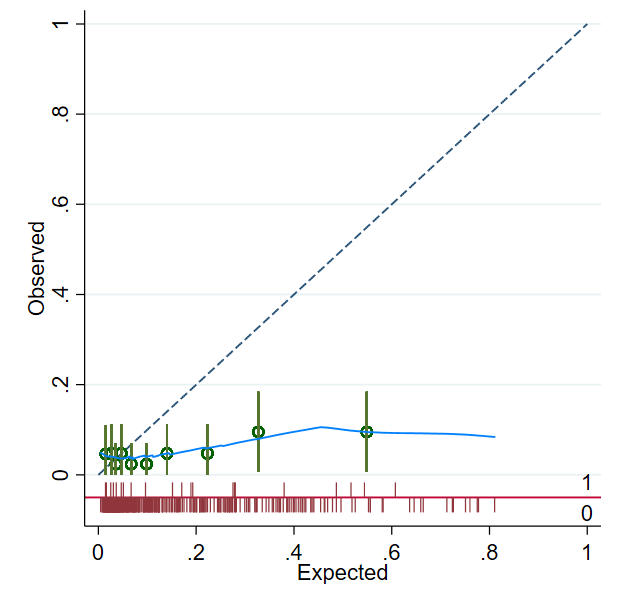 |
| --- | --- |
| STEMS  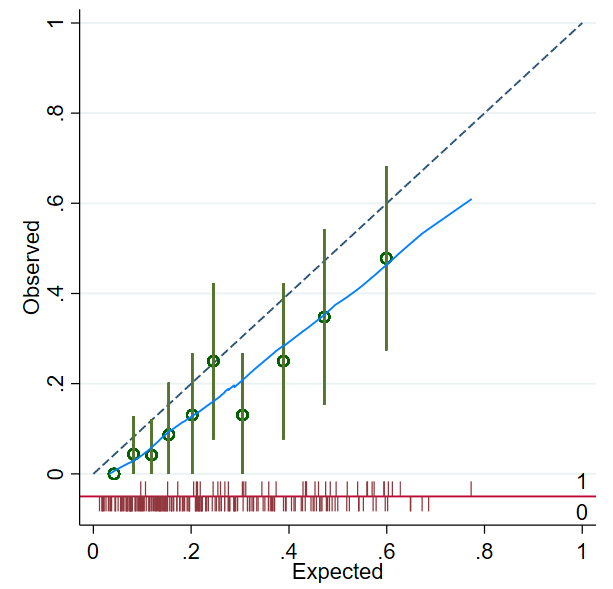 | STarT MSK-MT  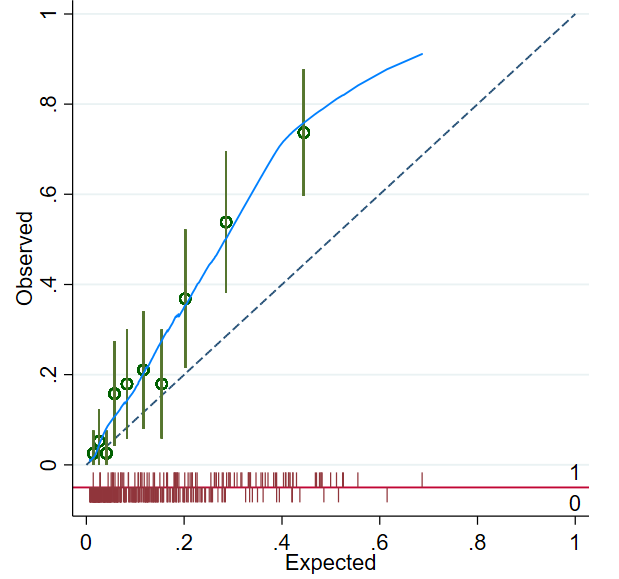 |
| STarT MSK-pilot  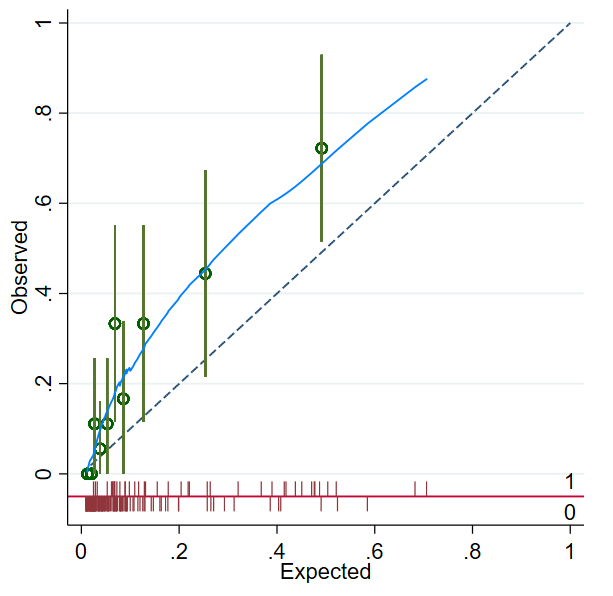 |  |

## Figure S5b: External calibration of 6-month presenteeism model (after adjustment for overfitting), for each study in the IECV cycle in which it was excluded from model development

| STEMS 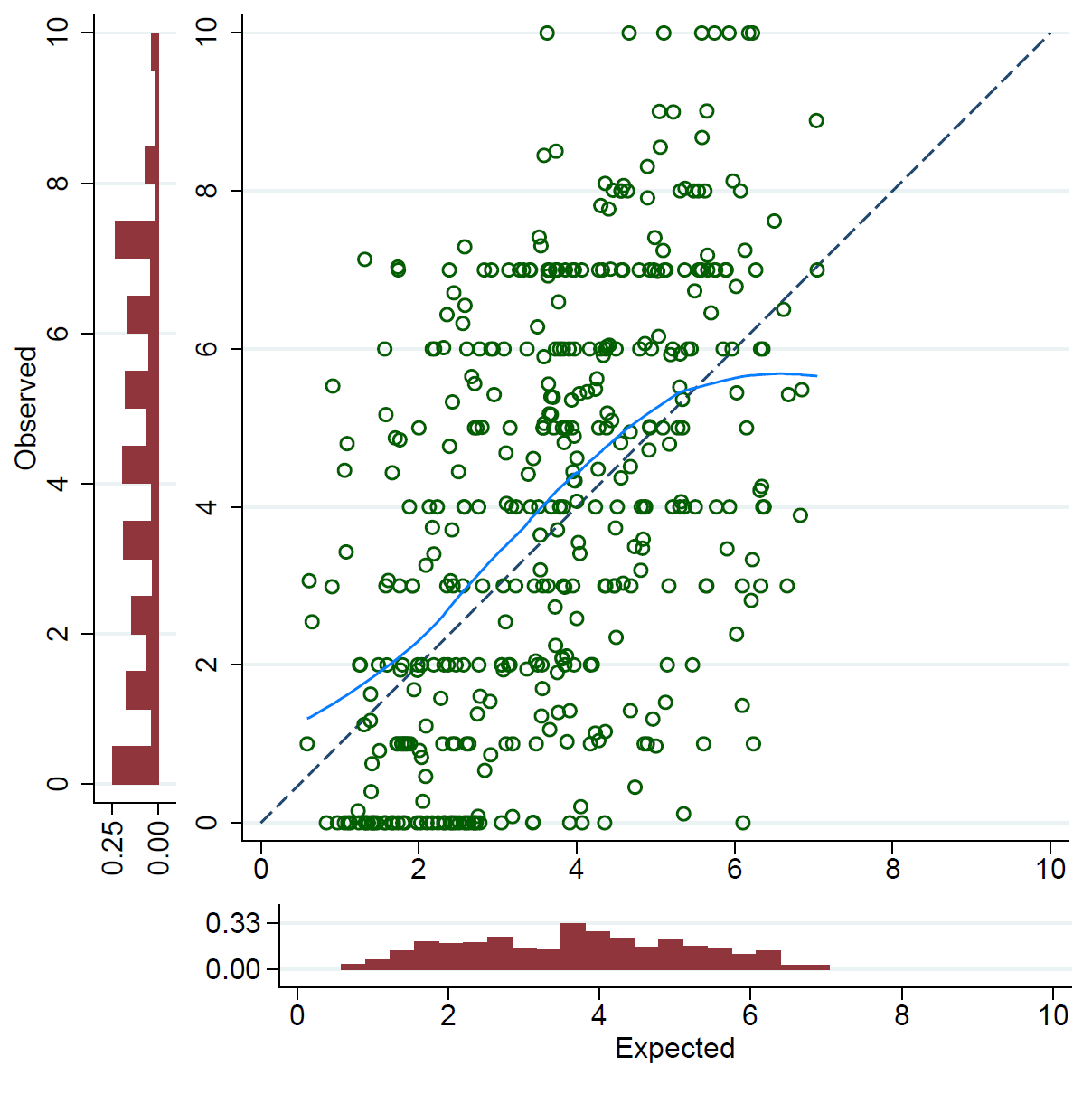 | STarT MSK-MT  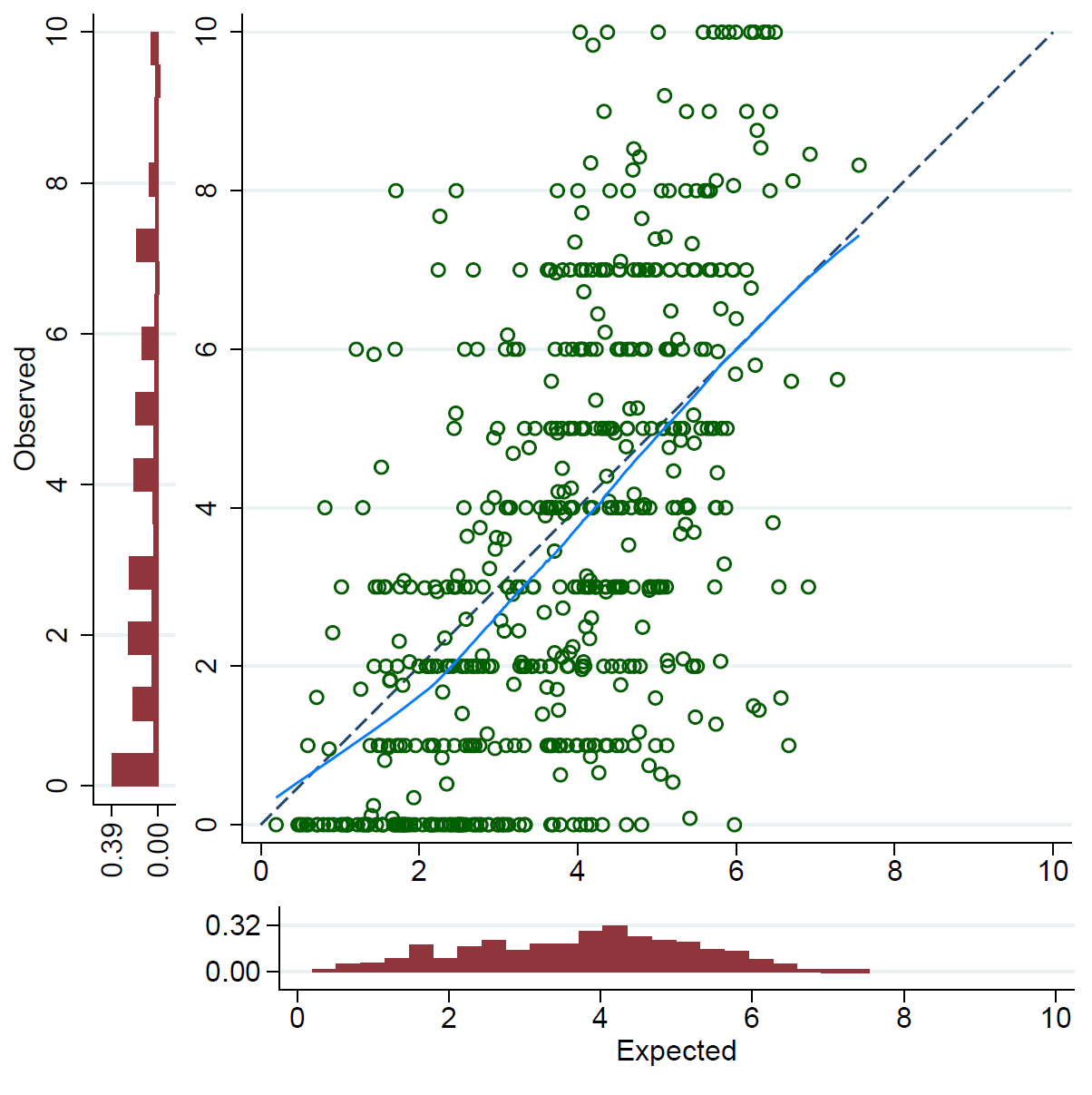 |
| --- | --- |
| STarT MSK-pilot  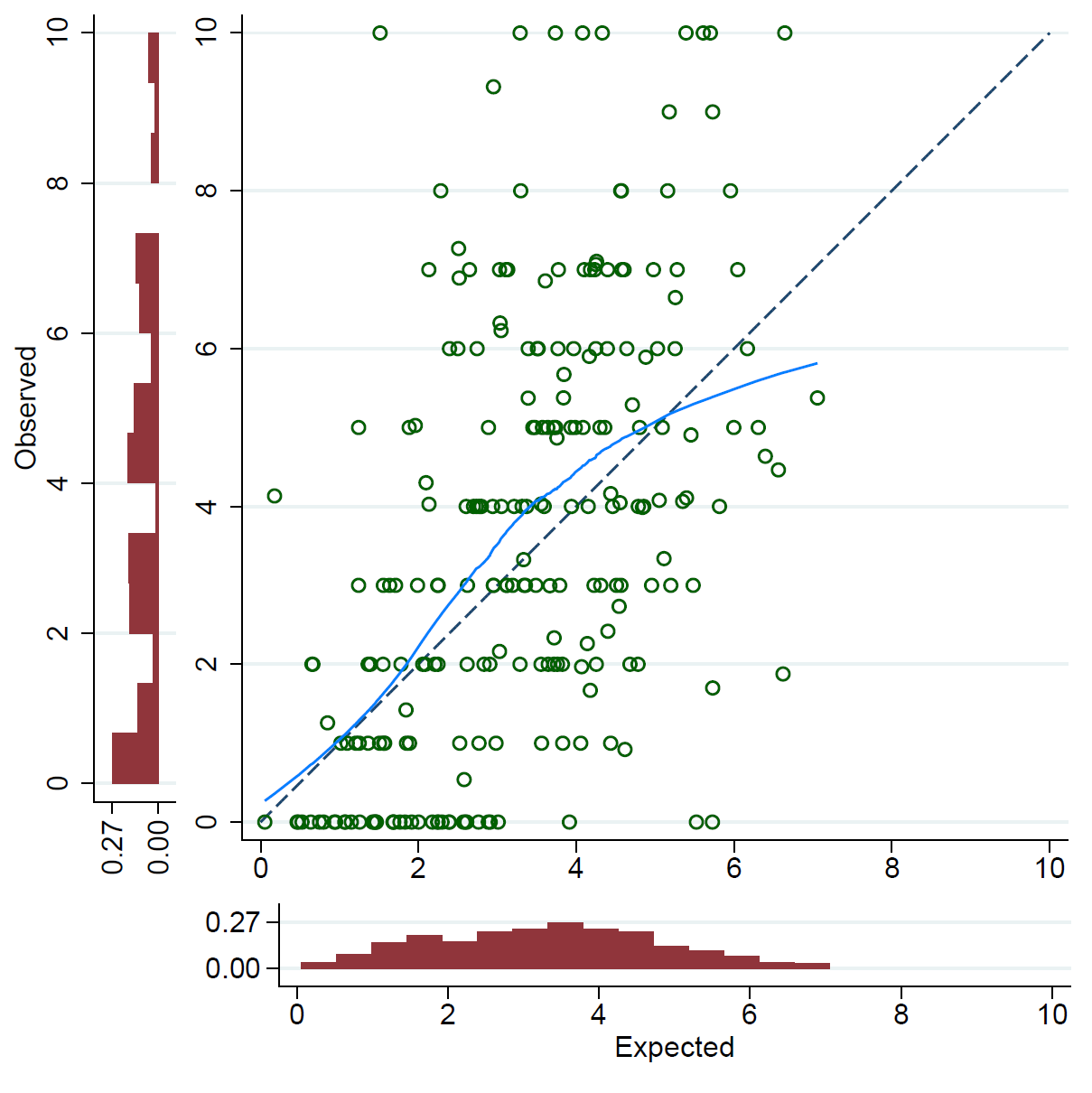 |  |

# References

1. Riley RD, Snell KI, Ensor J, Burke DL, Harrell FE, Jr., Moons KG, et al. Minimum sample size for developing a multivariable prediction model: PART II - binary and time-to-event outcomes. Stat Med. 2019;38(7):1276-96.

2. Roelen C, Thorsen S, Heymans M, Twisk J, Bültmann U, Bjørner J. Development and validation of a prediction model for long-term sickness absence based on occupational health survey variables. Disability and rehabilitation. 2018;40(2):168-75.

3. Riley RD, Snell KIE, Ensor J, Burke DL, Harrell Jr FE, Moons KGM, et al. Minimum sample size for developing a multivariable prediction model: Part I – Continuous outcomes. Statistics in Medicine. 2019;38(7):1262-75.

4. Riley R, Tierney J, Stewart LE. Individual Participant Data Meta-Analysis: A Handbook for Healthcare Research. Chicester: Wiley; 2021.

5. Carpenter JR, Kenward MG. Multilevel Multiple Imputation. Multiple Imputation and its Application2013. p. 203-28.

6. Quartagno M, Grund S, Carpenter J. jomo: A Flexible Package for Two-level Joint Modelling Multiple Imputation. The R Journal. 2019;11(2):205-28.

7. Vergouwe Y, Royston P, Moons KG, Altman DG. Development and validation of a prediction model with missing predictor data: a practical approach. J Clin Epidemiol. 2010;63(2):205-14.

8. White IR, Royston P, Wood AM. Multiple imputation using chained equations: Issues and guidance for practice. Stat Med. 2011;30(4):377-99.

9. Rubin D. Multiple Imputation for Nonresponse in Surveys. Wiley J, editor1987.

10. Vickers AJ, Van Calster B, Steyerberg EW. Net benefit approaches to the evaluation of prediction models, molecular markers, and diagnostic tests. Bmj. 2016;352:i6.

11. Roelen CA, van Rhenen W, Groothoff JW, van der Klink JJ, Bültmann U, Heymans MW. The development and validation of two prediction models to identify employees at risk of high sickness absence. European Journal of Public Health. 2012;23(1):128-33.
